# Protein conformational flexibility modulates kinetics and thermodynamics of drug binding

**DOI:** 10.1038/s41467-017-02258-w

**Published:** 2017-12-22

**Authors:** M. Amaral, D. B. Kokh, J. Bomke, A. Wegener, H. P. Buchstaller, H. M. Eggenweiler, P. Matias, C. Sirrenberg, R. C. Wade, M. Frech

**Affiliations:** 1 iBET - Instituto de Biologia Experimental e Tecnológica, Oeiras, 2780–157 Portugal; 2 Molecular Interactions and Biophysics, Merck KGaA, Darmstadt, 64293 Germany; 3 Molecular and Cellular Modeling Group, Heidelberg Institute for Theoretical Studies, Heidelberg, 69118 Germany; 4 Molecular Pharmacology, Merck KGaA, Darmstadt, 64293 Germany; 5 Medicinal Chemistry, Merck KGaA, Darmstadt, 64293 Germany; 6 ITQB - Instituto de Tecnologia Química e Biológica António Xavier, Universidade Nova de Lisboa, Oeiras, 2780-157 Portugal; 7 Cellular Pharmacology - Oncology, Merck KGaA, Darmstadt, 64293 Germany; 8 Zentrum für Molekulare Biologie, DKFZ-ZMBH Alliance, Heidelberg University, Heidelberg, 69120 Germany; 9 Interdisciplinary Center for Scientific Computing, Heidelberg University, Heidelberg, 69120 Germany; 10 Present Address: Sanofi-Aventis Deutschland GmbH, R&D, Biologics Research/Protein Therapeutics, Frankfurt am Main, 65926 Germany

## Abstract

Structure-based drug design has often been restricted by the rather static picture of protein–ligand complexes presented by crystal structures, despite the widely accepted importance of protein flexibility in biomolecular recognition. Here we report a detailed experimental and computational study of the drug target, human heat shock protein 90, to explore the contribution of protein dynamics to the binding thermodynamics and kinetics of drug-like compounds. We observe that their binding properties depend on whether the protein has a loop or a helical conformation in the binding site of the ligand-bound state. Compounds bound to the helical conformation display slow association and dissociation rates, high-affinity and high cellular efficacy, and predominantly entropically driven binding. An important entropic contribution comes from the greater flexibility of the helical relative to the loop conformation in the ligand-bound state. This unusual mechanism suggests increasing target flexibility in the bound state by ligand design as a new strategy for drug discovery.

## Abstract (web summary)

An understanding of the dynamics of drug binding and unbinding processes is important for drug discovery. Here, the authors give insights into the binding mechanism of small drug-like molecules to human Hsp90 by combining thermodynamics and kinetics studies as well as molecular dynamics simulations.

## Introduction

Understanding protein–drug binding mechanisms, and characterizing their thermodynamics and kinetics are fundamental prerequisites to developing effective drug discovery procedures and, indeed, to developing effective drugs. It has been demonstrated that the duration of the pharmacological action^1–4^ of a drug molecule is frequently related to its target residence time, *τ* (= 1/*k*_off_, where *k*_off_ is the rate constant for dissociation of the drug–target complex), rather than its binding affinity (which determines the equilibrium dissociation constant, *K*_D_)^5,6^. Moreover, drug–target binding kinetics (characterized by association, *k*_on_, and dissociation, *k*_off_, rate constants) have been shown to be relevant for predicting drug efficacy and off-target toxicity^1,7^.

**Fig. 1:**
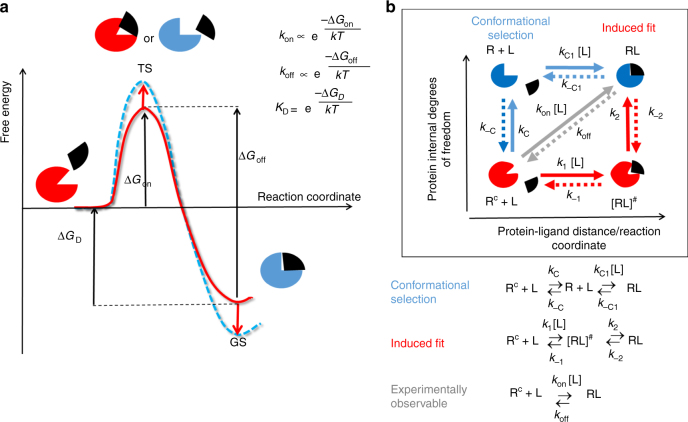
Models of drug–target binding. **a** Schematic diagram of a one-barrier drug–target binding free energy profile. A one-step model with one free energy barrier is used to derive the experimental rate constants. The figure and equations show how the steady-state rate constants relate to the free energy differences shown. The residence time of a drug bound to its target, *τ* (which is the reciprocal of the rate constant for dissociation of the drug–target complex, *k*_off_), results from the “difference” in free energy between the transition state (TS) and the bound ground state (GS), Δ*G*_off_. The red arrows indicate that prolongation of the *τ* can be achieved by stabilizing the GS (increasing the magnitude of Δ*G*_D_), destabilizing the TS (increasing Δ*G*_on_) or a combination of both (i.e., $K_{{\rm {off}}} \propto {e}^{\frac{{ - \Delta G_{{\rm {off}}}}}{{kT}}} = K_{\rm {D}}{e}^{\frac{{ - \Delta G_{{\rm {on}}}}}{{kT}}}$). **b** Diagram schematically illustrating different mechanisms of drug binding involving protein conformational changes. R and R^C^ denote two different conformations of the protein, the latter requires conformational changes for ligand binding. These may occur by conformational selection (blue path) or by induced fit upon formation of an encounter complex [RL]^#^ (red path), or by a combination of the two mechanisms. Binding proceeds through an energetically unfavorable intermediate state (TS in panel A or a local minimum in a 2 (or more)-step binding free energy profile) that, in the conformational selection and induced fit mechanisms, corresponds, respectively, to the R+L or [RL]^#^ state of the system); the final complex is denoted by [RL]. *k*_C/–C_ and *k*_2/−2_ are the rates of intrinsic and ligand-induced protein conformational transitions, respectively; *k*_1/−1_ and *k*_C1/−C1_ are rates of formation of the bound and encounter complexes, assuming that the protein is in conformations R and R^C^, respectively; *k*_off_ and *k*_on_ are experimentally observed off- and on–binding rates. The gray path and third equation describe the pseudo-one-step binding process shown in (**a**) is used to derive the experimental rate constants

A commonly used strategy in drug design is to modify a lead compound to increase the binding affinity (i.e., minimize *K*_D_) by stabilizing the bound ground state, denoted GS in Fig. 1a, which shows a simple 1-barrier free energy profile for binding. However, an improvement in binding affinity does not necessarily lead to a prolongation of the residence time, since the ground state stabilization may be compensated by stabilization of the transition state (TS in Fig. 1a). On the other hand, *τ* can be increased by destabilizing the transition state, and simultaneously slowing the association rate, while preserving the same binding affinity. In one of the very few publications on this topic, it was shown that both transition state destabilization and ground state stabilization contributed to the prolongation of the residence times of 27 drugs and inhibitors of various enzymes. However, the underlying mechanisms of transition state stabilization or destabilization are not well understood^8^. Perhaps the most compelling evidence of the influence of transition state destabilization in the modulation of residence time comes from a recent study carried out by Spagnuolo et al., in which they developed triazole-containing diphenyl ether compounds with increased residence times on InhA and slower association rates but little changed binding affinities^9^.

From a thermodynamic perspective, the early stages of ligand design and optimization typically focus on drug–target interactions in the binding site and their enthalpic optimization^10^. Entropy is primarily considered in terms of solvation of hydrophobic groups, while target flexibility is rarely taken into account. The generally rather static view of the binding event has been put into question by several studies of the contribution of target conformational entropy to the free energy of protein–ligand association^11–17^. Nonetheless, analysis of various databases^18^, with data from isothermal titration calorimetry (ITC) measurements on the thermodynamics of ligand binding to proteins confirms that strong entropic binders driven by conformational flexibility are still hardly represented^19^.

Binding kinetics and thermodynamics can also be affected by protein flexibility, but this aspect has only been addressed sporadically in the literature^13,20,21^. Analysis of enzyme inhibition kinetics reported in ref. ^22^ suggests that slow inhibition is often associated with a two-step binding process, where the first step of fast binding to an intermediate state is followed by a slow protein conformational adjustment, leading to a final bound complex. In contrast, Pargellis et al. have shown that the origin of the slow binding kinetics of a diaryl urea class of allosteric highly potent inhibitors against p38 MAP kinase is due to the large conformational change of the DFG motif, which occurs only rarely in the protein apo state, gating selection of a protein conformation compatible with inhibitor binding^20^. These examples represent two main approaches to describing the contribution of the target flexibility to molecular recognition: induced-fit^23^ and conformational selection^24^, respectively (Fig. 1b). However, the majority of protein–ligand-binding events cannot be exclusively assigned to one of these models and are likely to involve both mechanisms, with conformational selection and induced adjustments promoting complex formation^25,26^.

Here we report the investigation of the role of protein flexibility in the modulation of binding kinetics and equilibrium thermodynamics of drug binding to heat shock protein 90 (HSP90). HSP90 is one of the most abundant proteins in the cytoplasm of eukaryotic cells, comprising 2–5% of cytosolic protein under non-stressed conditions^27^. It is a ubiquitous molecular chaperone intimately involved in a variety of pathways, including cell signaling, proliferation and survival, and protein folding. It has emerged over the last 25 years as a potential target for the treatment of cancer^28–33^. HSP90 forms flexible homodimers, where each monomer consists of three domains that are linked by flexible regions^31,34–36^. The N-terminal domain of HSP90 (N-HSP90) contains the nucleotide-binding site, which has been the target site for drugs interfering with ATP binding and ATPase activity.

**Fig. 2:**
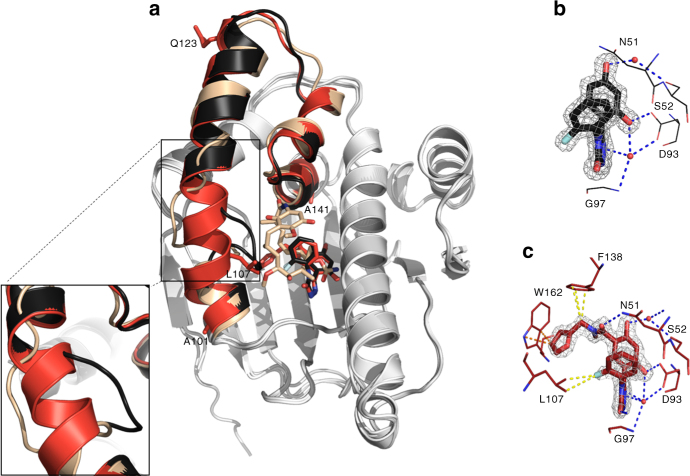
Comparison of different conformations of N-HSP90. **a** Overlay of three N-HSP90 crystal structures in complex with compounds **1** (black), **20** (red) and geldanamycin (wheat, PDB 1YET), representing loop-in, helical and loop-out conformations, respectively. The protein structures are shown in gray except α-helix3 (residues 101–123) and the lid segment (residues 107–141). A detailed view of the different conformations of α-helix3 is given in the inset. **b** Protein-ligand interactions representative of the loop-in conformation (compound **1**). **c** Protein-ligand interactions representative of the helical conformation (compound **20**). Dashed lines indicate interactions (blue: hydrogen bonds, yellow: aromatic, brown: hydrophobic). 2F_o_-F_c_ electron density maps, contoured at 1.5*σ*, are shown in gray around each ligand

The unbound N-HSP90 has a highly flexible lid segment comprising residues 107–141^37^. Structures of N-HSP90 in the apo-form and complexed with a variety of small molecules show remarkable plasticity, particularly in residues 104–111 located in α-helix3, that adopt “loop-in” or “loop-out” conformations^38^ (Table 1, Fig. 2). More recently, crystal structures have revealed ligands occupying an additional binding subpocket created by the rearrangement of residues 104–111 into a continuous helical conformation^39–42^ (Fig. 2; compounds bound to helical and loop-in conformations will be referred hereafter as “helix-binders” and “loop-binders”, respectively). Significantly, different thermodynamic profiles were observed for the binding of different molecules to N-HSP90. Specifically, the entropic contribution to binding may be unfavorable (as for ADP^43^) or favorable (as for AMPPCP^37,44^ and macrocyclic compounds^43^), suggesting different effects of local protein conformations.

**Table 1:** Chemical structure of compounds 1–20 with R^1^ and R^2^ substitutions

Loop binders are numbered **1**–**6**, helix-binders are numbered **7**–**20**

The mechanism by which N-HSP90 flexibility affects small molecule binding is not completely clear. Several studies have shown that the molecular recognition and binding of the flexible lid segment with different substrates and inhibitors is likely to involve both induced-fit and conformational selection mechanisms. Whereas Gooljarsingh et al. demonstrated that the binding of geldanamycin to Hsp90 is indicative of a two-step binding model with induced-fit^44^, equilibrium and kinetic studies by Onuoha et al. on the geldanamycin derivative 17-DMAG binding to HSP90 are consistent with a single-step process^34^. NMR^37^ and MD^45^ studies provide further evidence that the N-terminal domain of Hsp90 in its free state may exist in different conformations, with the lid segment undergoing internal motions on the µs–ms time scale, and the ligand-binding event modifying the population distribution towards more stable conformations.

The remarkable flexibility of the binding pocket of N-HSP90 was the decisive factor in leading us to investigate the kinetics and thermodynamics of inhibitor binding in more detail, as well as the impact of this plasticity on the common understanding of drug–target interactions. Moreover, it has been demonstrated in previous studies that tumor reduction directly relates to the level of HSP90 occupancy^46^. Importantly, the degradation of HSP90 client proteins was observed even after the drug had been cleared from the plasma and significantly reduced in the tumor, which clearly shows the importance of a slow inhibitor unbinding rate.

Here we report a detailed experimental and computational characterization of the binding properties of 20 members of a resorcinol class of HSP90 inhibitors (Table 1). We found that the inhibitors display a diverse range of kinetic and thermodynamic profiles. We carried out mutagenesis experiments to gain insights into the effects of protein flexibility on ligand binding. We discovered high-affinity compounds with long-residence times, whose binding is mainly entropically driven and is favored by the conformational flexibility of the ligand-bound protein. This study reveals an unusual mechanism of binding of small molecules to a protein target, which can be considered along with other strategies in drug design projects.

## Results

### Crystal structures of ligand-free and bound forms of N-HSP90

For 7 of the 20 compounds, it was possible to obtain crystal structures of the complexes bound to N-HSP90 with loop-in (**1**,** 6**) and helical (**8**, **14**, **16**, **18**, and **20**) conformations of the α3-helix region (loop- and helix-binders, respectively; Supplementary Table 1). All ligands occupy the ADP-binding site and form typical interactions, which include hydrogen bonds of the 2-hydroxyl group of the resorcinol ring with the side chain of D93 and with a water molecule that generally mediates interactions with G97 (Fig. 2a and Supplementary Fig. 1). The 4-hydroxyl group of the resorcinol ring forms a hydrogen bond with S52 through another ordered water molecule. The main difference between the structures is the conformation of residues 104–111, which can adopt either helical or loop-in conformations. In complexes with compounds whose R^1^ substituent exceeds one atom (compounds **8**, **14**, **16**, **18**, and **20**), these residues have the helical conformation with an adjacent hydrophobic pocket lined by the side chains of M98, L107, F138, Y139, V150, and W162. Considering their R^1^ substituent, compounds **9**–**11**, **13**–**17**, and **19** were also expected to form complexes with residues 104–111 of N-HSP90 in the helical conformation and were therefore assigned as helix-binders. Steric clashes between the ligands and residues 105–107 would prohibit these complexes from existing in a loop-in conformation (Supplementary Fig. 2). Similarly, compounds **2**–**5** were assigned as loop-in binders based on the similarity in size of their R^1^ substituent to compounds **1** and **6**, for which crystal structures were determined.

In agreement with previous studies^38^, unbound N-HSP90 has a loop-in conformation in the α-helix3 region. As previously mentioned in the Introduction, it is likely that the N-terminal domain of Hsp90 may exist in a combination of different conformational states in its free state. The binding of a specific compound may alter the protein conformational distribution by increasing the population of states that are rarely observed (i.e., much less populated) in the unbound state. The fact that the helical conformation has not been observed in crystal structures of apo N-HSP90 indicates that this conformational state might exist as a transient conformation with a higher free energy and therefore be less populated.

### Thermodynamic profiles of N-HSP90 inhibitors

**Fig. 3:**
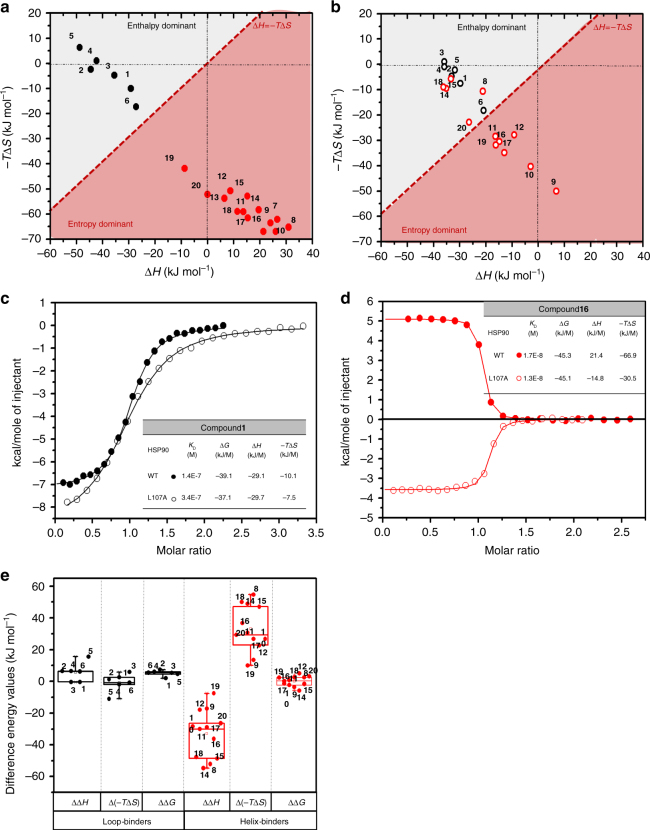
Thermodynamic profiles of N-HSP90 inhibitors measured by ITC. The enthalpic and entropic components of the binding free energy are shown in **a** and **b** for for WT N-HSP90, and the L107A mutant, respectively. The dashed diagonal line (Δ*H*=−*T*Δ*S*) divides the plot into two main areas where the enthalpy (gray) or the entropy (red) dominate the binding free energy (Δ*G*). Compounds **1**–**6** are loop binders and are colored black and compounds **7**–**20** are helix-binders and are colored red. **c**, **d** Isothermal titration calorimetry fitting curves for compounds **1** (**c**) and **16** (**d**) bound to WT N-HSP90 (full circles) and to the L107A mutant (open circles) with thermodynamic parameters shown in the respective tables. **e** Box plot showing the difference in the enthalpy (ΔΔ*H*), entropy (Δ(−*T*Δ*S*)) and binding free energy (ΔΔ*G*) for L07A relative to WT for loop- (colored black) and helix-binders (colored red). The boxes denote the 25th and 75th percentiles and the error bars the 5th and 95th percentiles

The thermodynamic profiles of the binding of the 20 resorcinol ligands to N-HSP90 obtained by isothermal titration calorimetry (ITC) are depicted in Fig. 3 (see also Supplementary Fig. 3 and Supplementary Table 2) and quantify the energetic differences between two states in equilibrium (free state and bound state).

The variations amongst the compounds show enthalpy-entropy compensation^47,48^. Measurements under different buffer conditions to account for possible superposition of protonation effects on the recorded heat signal (Supplementary Fig. 4) indicate that the enthalpy-entropy compensation is a property of the complexes rather than solely a manifestation of the measurement procedure. Interestingly, there is a strong difference in the binding thermodynamics of loop-in and helix-binding compounds. Specifically, “loop-in” binding compounds are primarily enthalpically driven, with favorable or small entropic contributions. In contrast, for the helix-binding compounds, there is a large favorable entropic contribution to binding, and most of them have a binding enthalpy penalty. This switch in thermodynamic profile is thus directly related to the conformation of the α-helix3 region with compensating differences in enthalpy and entropy of about 60 kJ mol^−1^ between loop-in and helix*-*binders. To explore the mechanism underlying this effect, we evaluated the different entropy components that may contribute to the ligand-binding entropy.

**Fig. 4:**
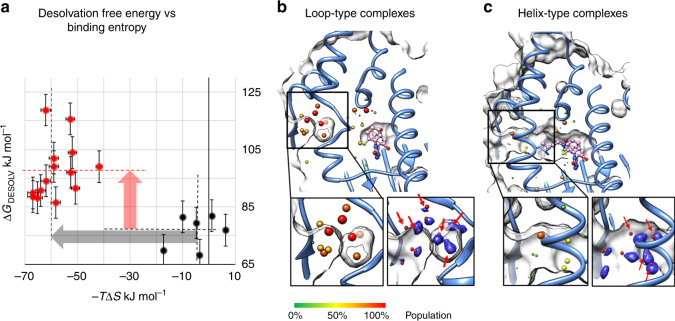
Simulation of the protein and ligand hydration effects. **a** Relation between the computed desolvation free energy of the inhibitors (see Methods section) and their measured binding entropy in ITC experiments. Compounds assigned as loop-binders are colored black and compounds assigned as helix-binders are colored red. Error bars show the root mean squared error of 3D-RISM predictions against experiment (RMSE=5.4 kJ mol^−1^ as reported in ref. ^50^). Black and red dashed lines indicate the average values of the desolvation energy and binding entropy for loop- and helix-binders, and the arrows show the corresponding differences between loop- and helix-binding compounds, as observed in experiment (gray) and in computations (light red). **b**,** c** Conserved water sites observed in loop-in (**b**) and helical (**c**) crystal structures (listed in Supplementary Table 5). The degree of conservation is visualized by increasing size and color; only water sites within 0.8 nm of N106 are shown. In the insets, water sites predicted by GIST^68^ are depicted by blue mesh iso-surfaces at a water density value twice that of bulk water; the oxygen atoms of the crystallographic water sites are represented by red spheres; red arrows indicate the positions of stable water sites predicted by 3D-RISM simulations^66^ (for details, see Supplementary Information)

Generally, the entropy change upon binding may arise from a reduction in translational and rotational degrees of freedom, an alteration of the conformational flexibility of the binding partners, and from the reorganization of their solvation shells upon binding. Favorable entropic contributions to the binding energy are usually considered to be driven by desolvation effects as the burial of hydrophobic surfaces leads to the displacement of structured water molecules into the bulk solvent^49^. We therefore first compared the solvation properties of the inhibitors and of the loop-in and helical conformations of N-HSP90. For uncharged compounds, desolvation is a hydrophobic effect that is predominantly entropic and thus the desolvation free energy can provide a good estimation of the entropy change due to the solvent. In Fig. 4a, computed desolvation energies are compared with experimental values of −*T*Δ*S* for all compounds studied (data are also given in Supplementary Table 4). Due to the generally larger size of the helix-binders in comparison with the loop-in-binding compounds, their desolvation is more energetically unfavorable. This trend is opposite to that for the binding entropies derived from ITC measurements.

Differences in the entropic contribution to binding energy of loop-in- and helix-binders may also arise from structural differences in the hydration shells of the loop-in and helical complexes, particularly because an additional hydrophobic pocket is formed in the latter case. To estimate the corresponding entropic difference, we compared the hydration shells of the two protein conformations. Since the nucleotide-binding pocket part is conserved in both loop-in and helical structures, we focused solely on the difference in the water molecules trapped on the protein surface around the flexible part of the α-helix3 region, whose entropy is much lower than in the bulk solvent. The positions of the stable water sites were evaluated from explicit solvent MD trajectories and compared with 3D-RISM^50^ computations based on the first-principle theory of solvation. Remarkably, both methods reveal very similar positions of the water sites around the flexible α-helix3 region, with no significant differences in the number of stable water sites being observed in the two protein conformations (4–7 sites, see Fig. 4b, c and Supplementary Table 5). The predicted water sites agree well with statistical analysis of the positions of water molecules in 16 PDB structures (8 from each conformational type of the complex as listed in Supplementary Table 6 and Supplementary Fig. 5c, d). However, the total number of water sites in loop-in crystal structures is larger than in helical ones (10 and 6, respectively; see Supplementary Table 5) albeit with a lower population of the former with respect to those in the hydrophobic pocket of the helix-type structures (see analysis in Supplementary Fig. 5e), which may level the solvent entropy for both types of structures. Thus, we can conclude that the difference between loop-in and helical conformations in just four stable water sites is the greatest possible and an upper limit of the corresponding entropic contribution to the binding energy difference for loop-in relative to helical structures can be estimated as 34 kJ mol^−1^ (a typical entropy penalty per trapped water molecule is about 8.4 kJ mol^−1^ ref. ^51^). This value is notably smaller than the difference between the average entropic binding energy term for loop-in- and helix-binders (about 60 mol^−1^, Fig. 3a) and thus cannot alone explain the large difference in the thermodynamic profiles of loop-in- and helix-binders.

**Fig. 5:**
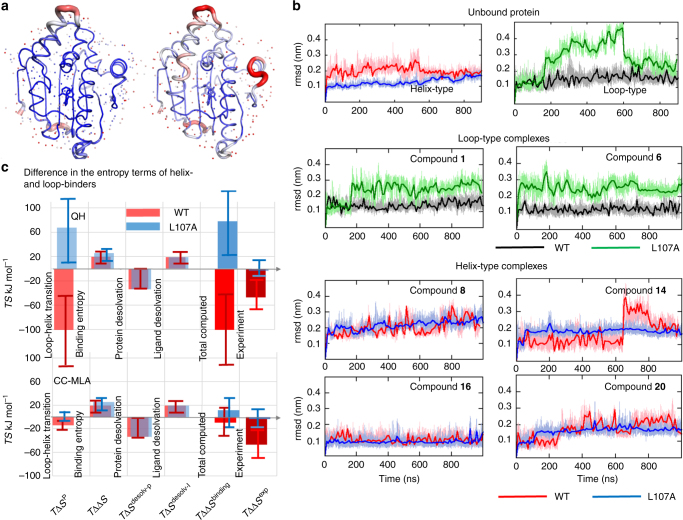
Simulations of the flexibility of WT-N-HSP90 and the L107A mutant. **a** Crystal structures of the loop-in (left) and helical (right) conformations with typical crystallographic B-factor values shown by cartoon ribbon radius and color, increasing from blue to red. **b** Variations of the RMSD of α-helix3 (residues 96–126) along 1 μs MD trajectories in loop-in and helical conformations of several complexes; the bold curves represent cubic splines of the original (gray, light-red, light-green, or light-blue) curves (the corresponding RMSF variations are shown in Supplementary Fig. 6). **c** Difference in the binding entropy of the helix*-* with respect to the loop-binders (averaged over complexes with compounds **8**,** 14**, **16**, **20**, and **1**,**6**, respectively) as observed in experiments (*T*ΔΔ*S* ^EXP^) and in computations (*T*ΔΔ*S* ^binding^) from the sum of the conformational entropy of the α-helix1 and α-helix3 segments (*T*Δ*S*_l-h_^P^ obtained using QH and CC-MLA approaches; see Methods section and Supplementary Figs. 7, 8b, and 9e); the binding entropy arising from the ligand and protein motion, but assuming that the protein has the same conformation in apo- and holo-states (*T*ΔΔ*S*, Supplementary Fig. 8b); the ligand desolvation energy (*T*ΔΔ*S* ^desolv-l^, Fig. 4a), and the protein desolvation energy obtained from the analysis of water sites around the flexible region of the α-helix3 in crystal structures (*T*ΔΔ*S* ^desolv-p^). Error bars indicate the standard deviations within an ensemble of complexes

Protein flexibility is another possible contributor to favorable binding entropy. As previously mentioned, the N-HSP90 crystal structures show higher B-factors for residues 103–111 (Fig. 5a) in the helical conformation, suggesting greater structural flexibility of this region compared to the loop-in conformation. Consistently, MD simulations of WT N-HSP90 reveal generally higher mobility of α-helix3 in the helical relative to the loop-in conformations (Fig. 5b and Supplementary Fig. 6a).

Generally, computation of the binding entropy from MD simulations requires sampling of both the bound and free states of the binding partners. However, the apo state of N-HSP90 may have different conformations that interconvert on the timescale of micro-seconds or longer. Sampling of such rare events is not computationally feasible. To overcome this limitation, one can approximate the difference in the binding entropy of loop-in and helix-binding compounds arising from the protein and ligand degrees of freedom as the sum of two terms (see details in Supplementary Fig. 7): (i) the difference between the ligand-binding entropy to the loop-in and helical- conformations, which includes the effect of the reduction of translational and rotational degrees of freedom, as well as of reduction of the ligand and protein (mainly side-chain) flexibility in a complex, assuming that the protein conformation is not changed upon binding; and (ii) the difference in the protein entropy in two conformations of the N-HSP90 complex. The first term was computed in a rigid rotor, harmonic oscillator approximation using the MM-PBSA program^52^. This part of the conformational entropy causes a binding energy penalty that is on average about 20 kJ mol^−1^ greater for the helix- than for the loop-binders (see average difference plotted in Fig. 5c and individual terms for each compound in Supplementary Fig. 8a), mainly due to the larger size and the greater flexibility of the helix-binders in solution.

Estimation of the entropy difference between the loop-in and helical conformations of the protein in the bound state is computationally very challenging because the long time-scale conformational changes should be taken into account. We used two computational methods that employ different approaches for reduction of the degrees of freedom to be sampled (see Methods section and Supplementary Methods for more details): (i) the rigid rotor quasi-harmonic, QH, approximation that usually provides an upper limit to the conformational entropy of global protein motions^53^, and (ii) the Correlation-Corrected Multibody Local Approximation (CC-MLA)^54^ method that yields the entropy of protein torsion angle degrees of freedom. Although the latter method is potentially more accurate, it does not take into account global backbone oscillations in the present model and tends to be negatively biased under sampling limitations and, therefore provides an estimate of the lower limit of the conformational entropy. Remarkably, both methods show a higher entropy of the helical conformation relative to the loop-in one, although the absolute values differ significantly (105 kJ mol^−1^ and 14 kJ mol^−1^ on average in binding free energy difference in the QH and CC-MLA simulations, respectively Supplementary Figs. 8b and 9). Summing up all the entropy terms discussed above gives a total estimated difference in the binding free energy of loop-in and helix-binders of about 101 and 10 kJ mol^−1^ for the QH and CC-MLA method used, respectively (see Fig. 5c). As upper and lower limits of the possible entropic contribution, these values are consistent with the average experimental value of 43 ± 10 kJ mol^−1^ for the same compounds (Fig. 5c).

These computations strongly suggest that the gain in entropy due to the switch of the protein upon ligand binding from the loop-in to the helical conformation can overcome the entropy loss due to complex stabilization as well as the contribution of desolvation effects to the entropy change.

### Kinetic behavior of N-HSP90 inhibitors and ex vivo efficacy

**Fig. 6:**
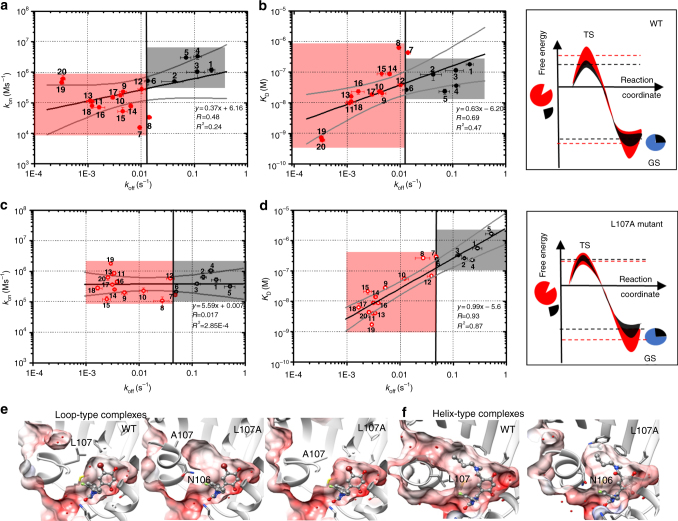
Contributions of thermodynamic affinity and association kinetics to the modulation of dissociation rate constants. **a**-**d** Logarithmic plots showing correlation of dissociation rate constant *k*_off_ (*x* axis) with the association rate constant, *k*_on_, and the dissociation constant, *K*_D_, (*y* axis) of compounds **1**–**20** determined by SPR for N-HSP90 WT (**a**, **b**) and L107A mutant (**c**, **d**). Points representing compounds assigned as loop-binders are colored black and compounds assigned as helix-binders are colored red. The black line is the linear regression with *R* ^2^ representing the coefficient of determination and *R* the correlation coefficient. The gray lines represent the 99% upper and lower confidence intervals. The error bars represent the standard deviation of at least three measurements. The red and black shaded regions highlight the different kinetic profiles of helix- and loop-binders, respectively. *k*_off_ is not strongly correlated either with *k*_on_ or with *K*_D_ for N-HSP90 WT. Thus, an increase of residence time is driven by a combination of GS stabilization and TS destabilization. For the L107A mutant, *k*_off_ is strongly correlated with *K*_D_ (*R*=0.69 for WT and *R*=0.93 for L107A) and not correlated with *k*_on_ (*R*=0.48 for WT and *R*=0.017 for L107A), indicating that residence time is mainly driven by GS stabilization. These relations are shown on the right in schematic pseudo 1-step free energy profiles for the binding reaction of helix- and loop-binders (shown in red and black, respectively; the filled area indicates the energy distribution among the entire compound series) Red and black dashed lines indicate average free energy values for the helix-and loop-binders, respectively. **e**, **f** The binding pocket shape observed in the crystal structures of N-HSP90 WT and L107A mutant for loop- and helical- complexes (**e**, **f**, respectively). Two alternative conformations observed in the crystal structures are shown for the L107A mutant co-crystallized with compound **6** in **e** The molecular surface of the protein is colored from red to white indicating increasing hydrophobicity

The results of binding kinetic measurements depicted in Fig. 6 (and given in Supplementary Table 3, see also Supplementary Fig. 10) reveal trends in the inhibitor binding characteristics that vary with the binding site conformation. The kinetic constants were derived assuming a 1-step model as illustrated in Fig. 1b, while multiple-step models could not be distinguished from the measured experimental data. The majority of the helix-binders (**7–20**) have lower association and dissociation rate constants for binding to WT N-HSP90 than the loop-binders (**1**–**6**, Fig. 6a), suggesting different binding mechanisms. Compounds that bind to the helical conformation can reach up to 650-fold slower dissociation rates when compared to compounds that bind to the loop-in conformation.

In order to identify the energetic factors involved in the residence time prolongation, it is important to recall at this stage that, in the one-step model used to derive the experimental kinetic constants (Fig. 1a), the kinetic barrier to dissociation can be increased by stabilizing the ground state enzyme-inhibitor complex (by increasing thermodynamic affinities), by destabilizing the transition state (by decreasing association rates) or by a combination of both. By plotting *k*_on_ (Fig. 6a) and *K*_D_ (Fig. 6b) against *k*_off_, we can see that overall, *k*_off_ shows a weak correlation with *K*_D_ and *k*_on,_ for WT N-HSP90 (slope and regression analysis in Fig. 6a, b), which indicates that *τ* prolongation is influenced by a combination of ground state stabilization and transition state destabilization.

While it is a common assumption that drug–target residence time prolongation can be achieved by maximizing drug–target affinity (ground-state stabilization of the enzyme−inhibitor complex), due to invariance of the *k*_on_, this study provides evidence that *k*_on_ can vary significantly, indicating transition state destabilization playing a role in prolongation of residence time. Helix-binders reach up to 230-fold slower association rates, when compared to loop-binders, indicating a higher transition state energy barrier for helix-binders, as depicted in the right-hand panel of Fig. 6.

Considering that the helical conformation of N-HSP90 is not observed in crystal structures of the unbound state, it is plausible to assume that protein dynamics, particularly the helix-loop transition, might play a major role in the modulation of the transition state. This conclusion is also supported by several studies that provide evidence that slow binding kinetics can be intimately connected to large conformational changes required for the binding of small molecules^20,22^.

The prolonged *τ* of the current resorcinol series was also reflected in a sustained inhibitory effect as measured by intracellular upregulation of HSP70 in A2780 cells, (Supplementary Table 7 and Supplementary Fig. 11). This observation is in agreement with the study performed by Tillotson et al., where HSP90 client protein degradation and cancer cell growth inhibition were demonstrated after incubation with the long residence time drug IPI-504^46^. These results provide further evidence that sustainable pharmacodynamics can be achieved by developing drugs with long residence times on their intended target.

### L107 is important for the high flexibility of α-helix3

Richter et al.^36^ demonstrated by three-dimensional NMR that the removal of the first 24 residues of yeast N-HSP90 increases the flexibility of the lid segment significantly, suggesting an interconnection of these two regions. Indeed, apo- and holo-crystal structures with the loop-in conformation confirm that the α-helix3 region forms several hydrogen bonds with α-helix1, particularly E25-K112 and Q23-N106 (Supplementary Fig. 12). In the helical structures, however, the Q23-N106 interaction is not possible due to the binding site rearrangement, and this may contribute to enhanced flexibility of the helical conformation of the α-helix3 region. Besides, NMR relaxation data on the AMPPCP-Hsp90 complex have shown a significantly high *J*(0) value for residues L107–K112 of HSP90 on the ms to µs time scale^37^.

Standard MD simulations also reveal high flexibility of the α-helix3 region (Supplementary Fig. 6a), but no helix-loop transition was observed on the µs time-scale. Therefore, we applied the L-RIP^55^ perturbation MD approach to investigate the stability of the α-helix3 interaction network. Perturbation of residues located in the lid-part of α-helix3 (residues 110-116) initiate the most pronounced conformational changes (Supplementary Fig. 13a), which indicates the general instability of this segment in agreement with experimental results for other organisms^56^. The rest of the α-helix3 (residues 103–109) is more stable; only perturbation of L107 induces notable distortions. Moreover, the replacement of the L107 side-chain by Ala leads to general stabilization of α-helix3 in the helical conformation in L-RIP simulations (Supplementary Fig. 13b), which suggests that L107 might play a major role in determining the structural and dynamic properties of α-helix3.

### The L107A mutation alters α-helix3 conformational stability

We determined crystal structures of the N-HSP90-L107A mutant in the apo-state and in complexes with representative loop- (**1**, **6**) and helix-binding (**14**,** 16, 20**) compounds. The crystal structure of the unbound L107A mutant (PDB code 5J80) closely resembles that of the WT apo-protein, with A107 occupying the same position as L107 in the WT (Supplementary Fig. 14). However, the complexes with the loop binding compounds **1** and **6** exist in two alternate conformations after L107A mutation: loop-in and loop-out (Fig. 6e), with increased mobility as indicated by higher local B-factors. The complexes of the mutant with helix-binding compounds revealed no significant differences when compared to the WT in terms of ligand-binding mode. However, the A107 side-chain does not occupy the position of L107, but is rotated towards α-helix1, forming an additional interaction between Q23 of α-helix3 and α-helix1 either through a water bridge or backbone interactions (Supplementary Fig. 12b) and allowing N106 to occupy the position of L107 in WT N-HSP90. Besides, the shape of the binding pocket is changed in the helical conformation, causing a partial closure of the transient hydrophobic pocket and consequently, a more compact and rigid conformation (Fig. 6f).

### Thermodynamic analysis supports key role of L107

ITC experiments for compounds **1**–**20** reveal different effects of the L107A mutation on the thermodynamics of loop- and helix-binders. Figure 3c, d demonstrate this for two representative loop- and helix-binders (compounds **1** and **16**, respectively). Although the binding affinity is marginally impaired for the N-HSP90-L107A-complex with compound **1**, the thermodynamic profile is very similar to that of the WT, with a major contribution of enthalpy to the binding. This suggests that loop-binders do not change the conformational distribution of the protein upon binding significantly, or that the thermodynamics of the bound- and unbound-states of the protein are similar (the available crystal structures suggest that the unbound state may be in either loop-in or loop-out conformations, see Fig. 2b). These results also support the hypothesis that interactions between α-helix1 and α-helix3 might be one of the main driving forces for the stability of the lid segment, as both conformations preserve the interactions between the two helices in WT and the L107A mutant (see Supplementary Fig. 12).

On the other hand, the thermodynamic profile of compound **16** binding to the L107A mutant is very different from that to WT (Fig. 3d). It binds to WT N-HSP90 in an endothermic reaction with a favorable entropic contribution and an enthalpic penalty, whereas the binding to N-HSP90-L107A is exothermic with a favorable enthalpy and a reduction of the favorable entropic contributions. This behavior was observed for all other helix-binders, with the exception of compound **9** (Fig. 3b), which suggests that the switch between the endo- and exothermic profile is largely driven by the conformational rearrangement of the protein due to the L107A mutation.

In agreement with these observations, the MD simulations of the complexes, as well as of the unbound protein, demonstrate less protein mobility in the helical conformation and strong destabilization of the loop-in conformations upon L107A mutation (shown in Fig. 5b, Supplementary Fig. 6). The latter effect leads to a switch from the loop-in towards an intermediate between loop-out and helical conformations in simulations (Supplementary Fig. 6d). Accordingly, the computed entropy of the complex in the helical conformation decreases, while that for the loop conformation increases, leading to a change in the thermodynamic profile of loop- and helix-binders upon mutation. This tendency is clearly observed regardless of the approach used for the entropy estimation (see Fig. 5c and Supplementary Figs. 8b and 9d showing the entropy of the loop-in and helical conformations of the protein obtained using QH and CC-MLA approaches). A change of the conformational stability upon mutation must also cause alteration of the conformational ensemble of the unbound protein in the direction of higher population of the helical conformation (even if such conformations exist only transiently) relative to the loop conformation, which should facilitate binding of the helix-binders. This is indeed the case as will be discussed below.

### The L107A mutation alters the transition barriers

The results of kinetic studies of the N-HSP90-L107A mutant binding to the same series of compounds depicted in Fig. 6, Supplementary Fig. 15 and summarized in Supplementary Table 3, show a systematic increase of dissociation rates upon protein mutation for the majority of the compounds, both helix- and loop-binders. Interestingly, association rates increase upon L107A mutation for the helix-binders, whereas for the loop-binders, the *k*_on_ values decreased (Fig. 6 and Supplementary Fig. 15). Consequently, the *k*_on_ values of the loop and helix-binders all fall in the same range, with similar transition state barriers (Fig. 6c).

As mentioned above, this result may be explained by the change in the conformational distribution of the unbound N-HSP90 upon mutation. If we assume a conformation selection model for the protein–ligand-binding event (Fig. 1b), a gain in stability of the helical conformation in the L107A mutant can be expected to cause an increase of its population in the apo-state and, as a result, a lower transition state barrier reflected by higher *k*_on_ values for all helix-binders (assuming that the ligand-binding process is faster than the transition between conformations). On the other hand, destabilization of the loop-in conformation in the L107A mutant may lead to a decrease of the *k*_on_ value for the loop-binders and consequently an increase in the transition barrier.

Overall, a strong correlation between *k*_off_ and *K*_D_ is observed in Fig. 6d (*R* = 0.92 and slope of linear regression 0.99), which indicates that the *τ* of compounds interacting with the L107A mutant is mainly driven by ground state stabilization, whereas the contribution from compound-dependent transition state destabilization is greatly reduced when compared to WT protein (*R* = 0.017 and slope of linear regression 0.007 for *k*_on_ and *K*_D_) (Fig. 6c). The latter observation suggests a reduction of the induced-fit effect that causes ligand-dependent association rates and an increase in the role of conformational selection. Identification of the binding mechanism, assuming that conformational selection and induced fit scenario can be clearly distinguished, would require experimental characterization of the changes in the pseudo-first-order rate constant *k*_obs_ with the compound concentration using stopped-flow kinetics and a multi-step model for analysis of the experimental data. Even in this case, however, as has been pointed out in ref. ^57^, unambiguous identification of the mechanism may not be feasible if *k*_off_ is slower than the rate of conformational transitions, which is the case for the compounds under study (taken into account that transitions between states in the unbound protein occur on the ms-μs time-scale^37^).

A detailed analysis of the thermodynamic properties that control the transition state using temperature-dependent Eyring analysis of SPR data would provide additional insights into the mechanism of ligand binding^58^ and will be the focus of further studies. Nevertheless, the data presented here, along with observations for other protein–ligand systems^1,2,8,9,21^, provide strong support for the key role that the conformational dynamics of ligand–protein complexes play in the modulation of the transition state and consequently in determining the drug–target residence time.

## Discussion

We have described an interdisciplinary study that elucidates the mechanism of drug binding to N-HSP90. We discovered long-residence time and high-affinity compounds with cellular efficacy, whose binding is mainly entropically driven. A key favorable entropic contribution arises from the conformational flexibility of the target in the bound state. These results illustrate that, although tighter interactions make binding more favorable, the thermodynamic signature of a strong binder does not have to be dominated by an enthalpic term. Particularly important for more disordered proteins^59^ is that increasing the entropic contribution of the protein in the ligand-bound state can provide an attractive optimization strategy in drug design, as expansion of the number of available conformational states in the bound complex promotes the binding event. Furthermore, this study clearly demonstrates the importance of combining thermodynamic and kinetic analysis for the understanding of the dynamics of drug binding and unbinding processes. Indeed, our analysis indicates that the compound-dependent protein conformations (loop-in and helical) in the bound state affect both equilibrium thermodynamics and binding kinetics. We find that compounds with entropically driven binding also have slow association and dissociation rates. This implies that tuning the protein conformational dynamics by ligand binding, e.g. by incorporating functional groups that make contacts that favor a more flexible conformation of the protein over a less flexible one, can provide mechanisms for modulation of the transition state for binding, and thus improving not only the binding affinity but also the lifetime of a protein–drug complex and consequently increasing drug efficacy.

## Methods

### Protein expression and purification

Human N-HSP90 WT and the L107A mutant were expressed and purified by Instituto de Biologia Experimental e Tecnológica and Proteros Biostructures GmbH, respectively.

Site-directed mutagenesis of Leu107 to Alanine (see Supplementary Table 8 for primers description) was performed with the Quick Change II XL from Agilent Technologies according to the manufacturer’s instructions.

Human N-HSP90 WT (NP_005339, α isoform 2) and L107A (aa 9–236) were cloned into pET28a including an N-terminal HIS-TEV-tag and expressed in *Escherichia coli* BL21 (DE3) RIL competent cells from Agilent Technologies, which were initially cultured in LB medium to OD_600_ of ∼0.5 at 37 °C, followed by additional growth while cooling to 18 °C to an OD_600_ of ∼0.8 before induction with 0.1 mM IPTG overnight. Cells were collected by centrifugation and resuspended in lysis buffer (2x Dulbecco´s PBS pH 7.4, 20 mM Imidazole, 10% glycerol, 1 mM TCEP, Complete tablets (Roche) and DNase). Cells were lysed by sonication and the cell lysate was cleared by centrifugation at 75,000 × g for 45 min at 4 °C.

The supernatant was loaded onto a Ni-NTA affinity column and the target protein was eluted in a linear gradient with buffer 2x Dulbecco´s PBS pH 7.4, 500 mM Imidazole, 10% glycerol, 1 mM TCEP. Peak fractions were desalted by dialysis and treated with TEV protease 50 U per mg protein at 4 °C overnight for histidine tag removal.

The cleaved proteins were separated by passing through* Ni-Sepharo*se resin and further purified using size-exclusion chromatography (Superdex S-200). The resultant pure recombinant HSP90 was stored at −80 °C in a buffer containing 30 mM HEPES/NaOH, pH 7.5, 150 mM NaCl, 1 mM TCEP, 10% Glycerol.

### Crystallization and structure determination

N-HSP90 WT (40 mg mL^−1^) was mixed in a 1:1 (v/v) ratio with the reservoir solution (H1 PACT: 100 mM Bis-Tris buffer pH 8.5, 20% PEG 3350, 200 mM NaF). Crystals were obtained using the hanging drop vapor diffusion method and equilibrating against 1 ml of the reservoir solution at 4 °C. For complex formation, selected compounds were soaked into crystals in a concentration range of 1–10 mM for one to seven days. The same conditions were used to obtain the structures of the L107A mutant. The resulting crystals were cryo-protected in mother liquor with 30% PEG 3,350, and flash-frozen in liquid nitrogen for synchrotron X-ray data collection.

All data sets were collected at 100 K on beamline SLS × 106 and processed with the XDS software package^60^. The structures were solved by molecular replacement, using BUSTER^61^. Model building was performed in Coot, with compounds and waters fitted into the initial |F_o_|–|F_c_| map, and the structures were refined using BUSTER. The coordinates of the apo and holo-structures of N-HSP90 have been deposited in the RCSB Protein Data Bank. The refinement statistics and PDB accession codes are given in Supplementary Table 1.

### Isothermal titration calorimetry

All the titration experiments were performed using the VP-ITC system from Malvern in 30 mM Hepes buffer pH 7.50, 150 mM NaCl and 0.5 mM TCEP. Protein was prepared by dialysis for 24 h against 1 liter of 30 mM Hepes pH 7.50, 150 mM NaCl and 0.5 mM TCEP. The final protein concentration in the sample cell as determined by the Bradford Assay was 50 μM. Ligand stock solutions of 10 mM in DMSO were diluted to 5 µM concentrations with ITC buffer and adjusted to 2% (v/v) DMSO. Both the titrate and titrant solutions were degassed prior to loading the calorimeter cell and injection syringe.

For buffer dependency titration experiments, buffer exchange to either Tris, Tricine or Pipes buffer was performed by dialysis. The same protocol was followed as previously described for compounds **1** and **16** (Supplementary Fig. 12).

The integrated heat data were fit with a one-site binding model using the Origin-7 software provided with the Malvern VP-ITC. Protein concentration was corrected by titration of a reference compound and normalized for the concentration of each ligand. All ligands bound to N-HSP90 have a stoichiometry of approximately unity.

### Surface plasmon resonance

SPR measurements were performed on a Biacore 4000 instrument from GE Healthcare. Recombinant N-HSP90 [His-Tev-huHsp90 (9–236)] was immobilized on a Biacore CM5 chip at 25 °C at a flow rate of 10 µL min^−1^ using amine coupling at pH 4.50 according to Biacore’s standard protocol. HBS-N (10 mM Hepes pH 7.40, 0.15 M NaCl) served as running buffer during immobilization. N-HSP90 was applied at a concentration of 20 µg.mL^−1^ in a buffer containing a 75-fold excess of 17-Dimethylaminoethylamino-17-demethoxygeldanamycin (17-DMAG). An unmodified carboxydextran matrix served as a reference surface. Hsp90 inhibitors stored as 10 mM stock solutions in 100% dimethyl sulfoxide (DMSO) were dissolved in running buffer (20 mM HEPES pH 7.50, 150 mM NaCl, 0.05% Tween 20, 1 mM DTT, 0.1 mM EDTA, 2% DMSO) and analyzed using two-fold dilution series. Kinetic titration experiments were performed at 25 °C with a flow rate of 30 µL min^−1^, a sample contact time of 120 s and a dissociation time between 300 and 600 s.

The data sets were processed and analyzed using the Biacore 4000 Evaluation software, version 1.1. Solvent corrected and double-referenced association and dissociation phase data were fitted to a simple 1:1 interaction model with mass transport limitations. Two state-reaction could not be distinguished from the measured experimental data.

The correlation of dissociation constants obtained from SPR and ITC experiments is given in Supplementary Fig. 16.

### Ex vivo HSP90 activity determined by Hsp70 upregulation

A2780 cells were plated in a volume of 180 µl in 96-well plates and incubated at 37 °C (in DMEM supplemented with 5% fetal bovine serum). 20 µl per well of medium including serial dilutions of the compounds were added to the culture plates and incubation was continued further for 24 h. The cells were fixed with formaldehyde for 30 min at room temperature. The plates were blocked for 1 h at room temperature with PBS containing 0.1% Triton® X-100 and 5% BSA and subsequently incubated with Hsp70 antibodies (Stressgene), anti mouse IgG-HRP and chemiluminescent substrate. All measurements were performed in duplicates.

### Chemistry

Information on the synthesis of chemical compounds is provided in WIPO (2006), WO2006/087077. Analytical data is provided in the Supplementary Note 1. ^1^H NMR spectra were recorded at 300 K unless otherwise specified using a Bruker Avance DPX 300, AV 400, DPX 500 spectrometer (TMS as an internal standard). 1 H NMR chemical shifts are reported in parts per million (ppm). ^1^H NMR data is reported as chemical shift (dH), relative integral, multiplicity (s = singlet, d = doublet, t = triplet, q = quartet, dd = doublet of doublets, ddd = doublet of doublet of doublets, dt = doublet of triplets, td = triplet of doublets, tt = triplet of triplets, qd = quartet of doublets) and coupling constant (J Hz). All of the compounds reported in the manuscript have a purity ≥ 95% unless noted otherwise.

### Structure preparation and explicit solvent MD simulations

Simulations were performed for compounds in complex with WT N-HSP90 and with its L107A mutant. For this, the crystal structures solved in the present study (**1**, **6**, **8**, **14**, **16**, and **20** for WT N-HSP90, and **16** and **20** for the L107A mutant) were employed. For simulations of the complexes of the L107A mutant with compounds **1**,** 6**, **8**, **14**, we generated starting structures from the crystal structures of the WT N-HSP90 by truncating the sidechain of L107 to alanine. Crystallographic water molecules located between the protein and the ligand as well as in binding sub-pockets at α-helix3 were explicitly included in starting structures for explicit solvent MD simulations. We employed Amber ff14SB^62^ and GAFF^63^ force fields for the protein and compounds, respectively. Atomic partial charges were computed for the ligands using the Restrained Electrostatic Potential (RESP) method as implemented in the R.E.D. webserver^64^. Two trajectories of about 1 µs with a step of 2 fs were simulated for each ligand using GROMACS 5.0.5^65^ software. Technical details of MD simulations are given in Supplementary Information.

### Perturbation implicit solvent MD simulations

A non-equilibrium MD method, Langevin-Rotamerically Induced Perturbation (L-RIP) approach^55^, was applied to explore the conformational flexibility of the α-helix3 region in N-HSP90. For these simulations crystal structures of the complexes of compound **16** and PDB 1YER (for a helix- and loop-type conformations, respectively) were employed. The L107A mutant structures were obtained by truncating the L107 side-chain to alanine. Ligand atoms and water molecules were removed. The structures were energy minimized, gradually heated to 300 K, and equilibrated, first under constant energy conditions (5 ps) and then with Langevin dynamics (damping coefficient of 10 ps^−1^). Then a L-RIP perturbation simulation was started: in each pulse, the total kinetic energy of a single residue in the binding site with a rotatable side chain was applied only to the rotational degree of freedom of the torsion angle, χ. A short 0.3 ps implicit solvent MD relaxation step with a Langevin thermostat (damping coefficient of 1 ps^−1^) was applied to let the excess kinetic energy in the side-chain rotation be transferred to nearby residues. This perturbation-relaxation procedure was repeated 1000 times for every rotatable residue in the α-helix3 site (K100-K116). The last snapshots of each pulse were combined into a L-RIP trajectory. 10 L-RIP trajectories were generated for each perturbed residue. The average RMSD of the residues in α-helix3 over all 10 trajectories for each perturbed residue was computed and is plotted in Supplementary Fig. 13.

### Computation of desolvation free energies of compounds

The desolvation free energy of a compound, was estimated as the hydration free energy taken with the opposite sign. Hydration free energies of all the compounds were computed using the version of the three-dimensional reference interaction site model, 3D-RISM^50^, as implemented in MOE^66^. The 3D-RISM method gives the spatial density of the solvent atoms in the presence of the solute potential described by a standard force-field (a 12–6 Lennard-Jones term and a Coulombic term with AM1-BCC semi-empirical charges were used in the present calculations). The solvation free energy comprises electrostatic and non-polar solvation components, where the latter were corrected in ref. ^50^ by introducing an additional scaled density correlation function with a scaling coefficient obtained by fitting the 3D-RISM solvation energy to the molecule solvation energy computed using free energy perturbation for a set of 504 organic molecules. The root mean squared error (RMSE) of the 3D-RISM solvation energies was 1.29 and 0.93 kcal mol^−1^ against experimental and free energy perturbation results.

### Protein hydration shell analysis using crystal structures

The water network around the flexible region of α-helix3 (residues 105–111) depends on its conformation and, therefore, may cause differences in the binding entropy. To estimate this difference, relative populations of water sites in crystal structures of complexes with loop and helix-binding compounds were analyzed using the WaTCH tool^67^ for a set of 16 crystal structures. Specifically, 11 PDB structures (Supplementary Table 6) with helical and loop-in conformations were selected from different studies to avoid bias in the assignment of water positions; additionally, 5 structures solved in the present study (for compounds **1**, **6**, **14**, **16**, **20**) were included in the set. The relative population density values computed with WaTCH for stable water sites that are close to the middle of α-helix3 (within 0.8 nm of N106) and that are different for complexes with loop and helix-binding compounds (shown in Fig. 4b, c) are summarized in Supplementary Table 5.

### Protein hydration shell analysis using MD simulations

The structural and thermodynamic properties of the water molecules were analyzed using the Grid Inhomogeneous Solvation Theory (GIST) method^68^ implemented in the Amber tools package^69^. In the GIST approach, an explicit solvent MD trajectory is used for mapping the water density onto a 3D grid, then the structural and thermodynamic properties of water are computed for each grid cell. For GIST analysis, the first 5000 snapshots from production trajectories starting from each of the PDB structures co-crystallized with helix- and loop-in conformations of the binding site, and superimposed using the protein backbone were used. The protein structure was not restrained, but was stable enough over a time-scale of 10 ns to obtain a reliable water distribution; continuation of the trajectory to 40 ns led to an ~2-fold decrease of the water site populations. For GIST simulations, a cubic grid of size 16^3^ Å^3^ and spacing 0.5 Å was centered on the mid-point of the L107 and L106 Cα atoms. A default reference density of TIP3P water molecules, 0.0329 molecules/Å^3^ in bulk, was used. Identified water sites situated either between α-helix1 and α-helix3 or between α-helix3 and the beta-strands beneath are given in Supplementary Table 5. We did not include water sites in the ATP-binding pocket since they are dependent on the ligand bound and do not represent a general difference between the helical and loop-in structures.

### Protein hydration shell analysis using 3D-RISM

3D-RISM analysis of the solvent density implemented in MOE software^66^ was carried out using available structures with helical and loop-in conformations of the binding site (**8, 14, 16, 20** and **1, 6**, respectively). The method provides occupancies and energies of the most energetically stable water molecules. The following parameters were used in the calulations: salt concentration of 100 mM, 9–6 Lennard-Jones potential with a cut-off of 12 Å, and default charge generation procedure; only water molecules associated with the positions of crystallographic water sites in the structures of the complexes were considered. The total number of stable water sites at the density iso-value of 1.5 found for helix-type complexes **8**, **16**, **14**, **18**, and **20** was 4, 6, 4, 6, and 6, respectively, and 5 for both loop-type complexes with compounds **1** and **6**. The computed binding free energies of the detected water sites for the compounds **16** and **1** are given in Supplementary Table 5 as an example.

### Computation of conformational entropy

The computation model for estimation of relative binding entropy is schematically illustrated in Supplementary Fig. 7. Specifically, the difference between the entropic contribution to the binding free energies of the *loop*- and *helix-binding* compounds was computed as the sum of two terms: *T*Δ*S*_l-h_^binding^ = *T*ΔΔ*S*_l-h_ + *T*Δ*S* ^P^_l-h_, where *T*ΔΔ*S*_l-h_ = *T*Δ*S*_l_ −*T*Δ*S*_h_ – is the difference in ligand binding free energy due to the change of ligand and protein conformational entropy, which arises from the short time-scale vibrational degrees of freedom of the protein (particularly, from side-chain motions), as well as from the loss of rotational and translational entropy of the ligand upon binding, and *T*Δ*S*_l-h_^P^ is the free energy term arising from the difference in entropy between the loop-in and the helical conformations of the protein. The choice of the computational method employed for estimation of the *T*ΔΔ*S*_l-h_ and *T*Δ*S* ^P^_l-h_ terms as well as limitations of the method employed in the present study are discussed in Supplementary Methods.

Particularly, we employed the harmonic (H) rigid body approximation and Normal Mode Analysis (NMA) as implemented in the MMPBSA.py script^52^ from Amber tools^69^ for the estimation of the Δ*S*_l_ and Δ*S*_h_ terms. This script provides the complete binding entropy as the difference between the entropies of the protein–ligand bound state and the free protein and ligand, including the rotational and translation entropy change upon complex formation. For NMA-H computations, 50 frames were extracted from the first 10 ns of the MD trajectories at equal time intervals. Since we do not have reliable information on the free state of the protein, we used the same snapshots for computing the entropy of both the free and the bound states of the protein and ligand by just extracting the corresponding binding partner from the structure. Mean binding entropy values computed for 50 frames and the standard deviation are shown in Supplementary Fig. 8a.

Two different methods, (1) quasi-harmonic approximation, QH, and (2) correlation-corrected multibody local approximations, CC-MLA^54^, were used for computing the difference in entropy between the loop-in and the helical conformations of the protein, *T*Δ*S* ^P^_l-h_. Computation of the protein entropy requires extensive sampling of multiple degrees of freedom and can be extremely computationally demanding. In the present case, however, the sampling space could be reduced. Indeed, there are several flexible elements of the protein structure and some of them, such as the protein termini and the C terminus of α-helix2, demonstrate motion in all complexes, which is not correlated with the motion of α-helix3 (vectors obtained from PCA are illustrated in Supplementary Fig. 6c, d), but make a sizeable contribution to the computed entropy values. To reduce the uncertainty of the entropy calculations, we considered only α-helix3 and α-helix1 segments (residues 96–126 and 36–41, respectively), whose motion is correlated and directly relevant to distinguishing between the conformational flexibility of the helical and loop-in structures. For estimating the entropy difference, we used an average value over complexes (either helix or loop-in type) employed in simulations (i.e., **1, 6, 8**, **16**, **14**,** 18**, and **20**).

For QH computations, we employed atomic coordinates of non-hydrogen atoms in a 1 µs MD trajectory. Four trajectory segments were analyzed (200–500 ns, 400–700 ns, 200–700 ns, and 100–800 ns with snapshots extracted at 20 ps intervals) to evaluate average entropy values and their standard deviation for each complex (Supplementary Fig. 8b). A covariance matrix was constructed and diagonalized using “g_covar” and entropy calculations were performed using the “g_anaeig” tools of Gromacs^65^ package.

For CC-MLA computations, we employed two 1 μs MD trajectories for each complex with snapshots extracted with a 4 ps stride, and torsion angles involving only heavy atoms were sampled. The computed entropy values as a function of the simulated trajectory length illustrated in Supplementary Fig. 9a, b show clear but slow convergence that is not completely reached even with 500,000 snapshots from two trajectories, each of 1 μs. Simulations at different thresholds were carried out (illustrated in Supplementary Fig. 9c, d) and the minimum value for a particular complex (see Supplementary Fig. 9e) was used for entropy estimation, as suggested by the authors of the method^54^.

### Data availability

The coordinates and structure factors have been deposited in the Protein Data Bank under accession codes: 5J2V, 5J64, 5J2X, 5J86, 5J2, 5J9X, 5J86, 5J20, 5J80, 5J8U, 5J8M, 5J6N, 5J6L, 5J6M. Other data are available from the corresponding authors upon reasonable request.

## Electronic supplementary material

Supplementary Information
