# Supplementary material for: Protein conformational flexibility modulates kinetics and thermodynamics of drug binding
Source: Nat Commun. 2017 Dec 22;8:2276. doi: 10.1038/s41467-017-02258-w (PMC5741624; doi:10.1038/s41467-017-02258-w)
Supplement: Supplementary file 1 — Supplementary Information [file 41467_2017_2258_MOESM1_ESM.pdf]

# Supplementary Figures

## Compound 1

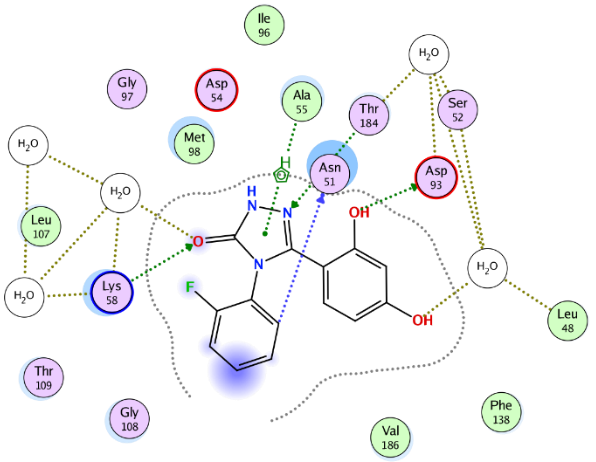


## Compound 6

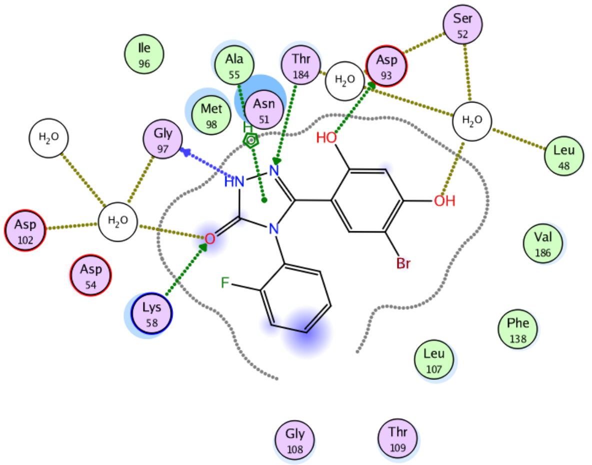


## Compound 8

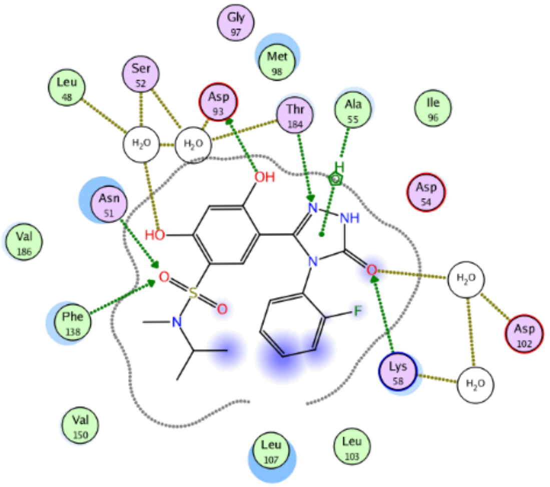


## Compound 14

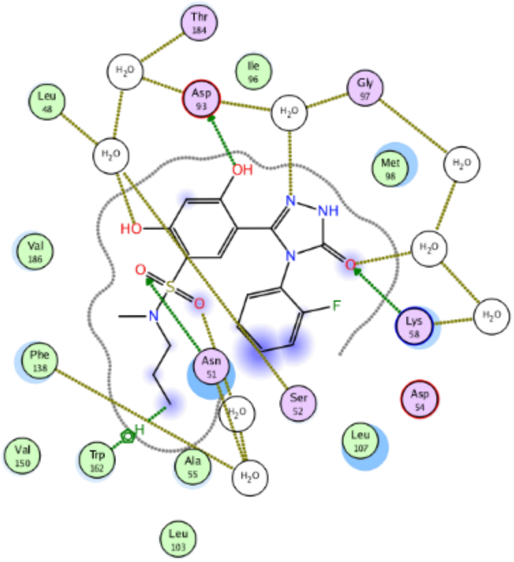

**Compound 16** **Compound 18**

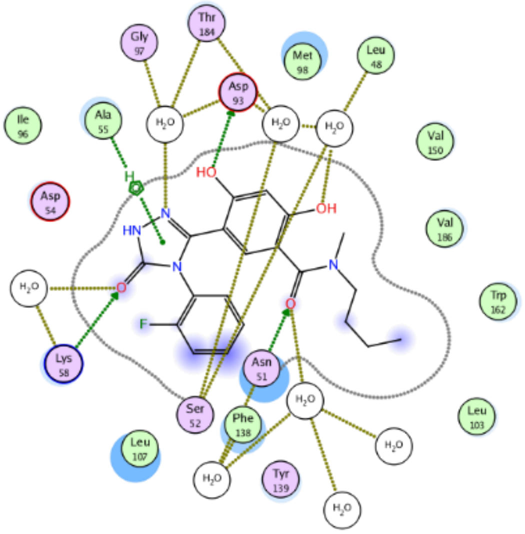


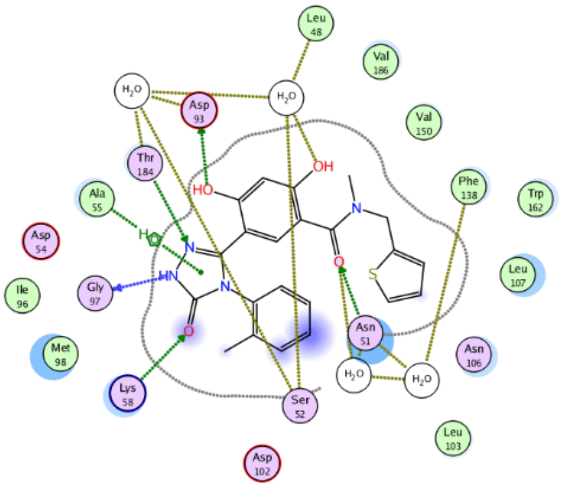


**Compound 20**

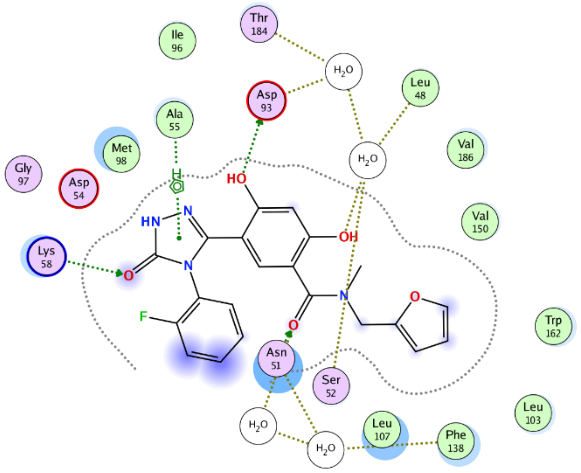


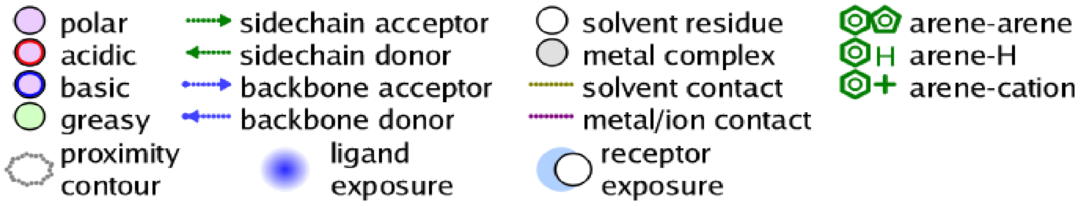


**Supplementary Figure 1** Two dimensional interaction diagrams of N-HSP90 with compounds **1**, **6**, **8**, **14**, **16**, **18** and **20** (PDB codes 5J64, 5J2X, 5J82, 5J27, 5J9X, 5J86, 5J20, respectively). Interaction diagrams were obtained by using the ligand interaction analysis feature of MOE.

A B
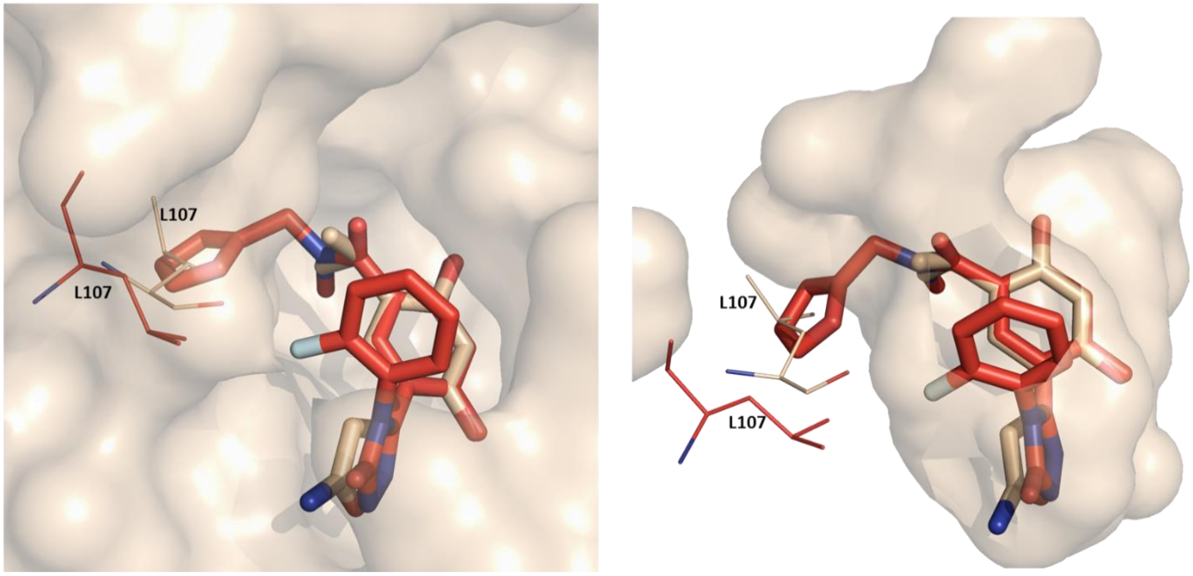


**Supplementary Figure 2** Steric clashes between the ligands and the N-HSP90 helical (**A**) and loop-in (**B**) conformations. Overlay of N-HSP90 crystal structures in complex with compound **20** (red) and HSP90 inhibitor FJ3 (**4-(5-amino-1,2-oxazol-3-yl)-6-(propan-2-yl)benzene- 1,3-diol**, PDB code: 4LWF, wheat), with residue 107 shown in stick representation. The molecular surfaces of the protein are displayed for the complexes with **20** (**A**) and with FJ3 (**B**). Residues 103-111 are in a helical conformation (**A**) and a loop-in (**B**) conformation, respectively. As shown on panel **A**, steric clashes between compound **20** (red) and residue 107 (wheat) would prohibit this complex from existing in the loop-in conformation observed in the N-HSP90-FJ3 complex. Considering the size of the R1 substituent compared to compound **20**, compounds **9**-**11**, **13**-**17** and **19,** for which the crystal structures were not solved, were assigned as helix-binding compounds. Considering the crystal structures determined for N-HSP90 bound to compounds **1** and **6**, and the fact that their R1 substituents do not exceed one atom in size, compounds **25** were assigned as loop-in binders.

**Compound 11**

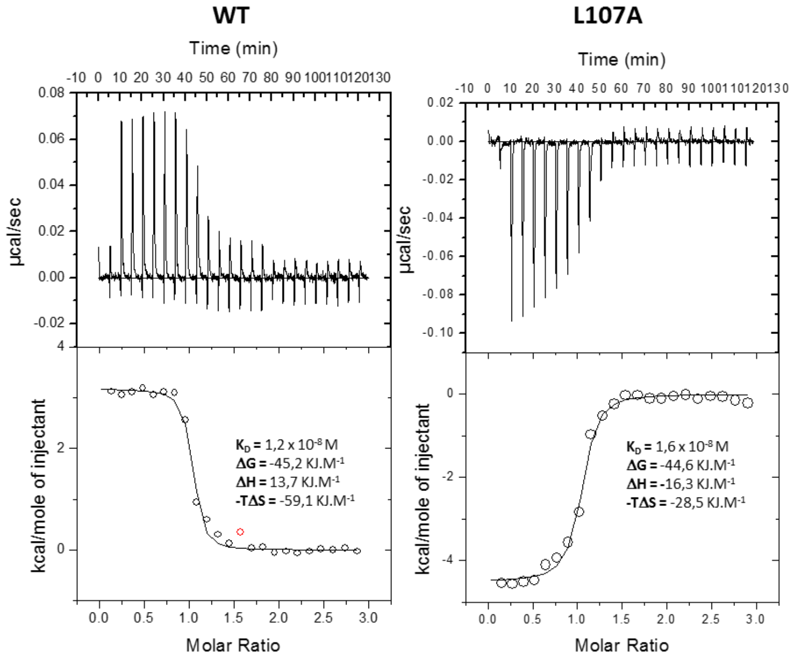


**Compound 2**

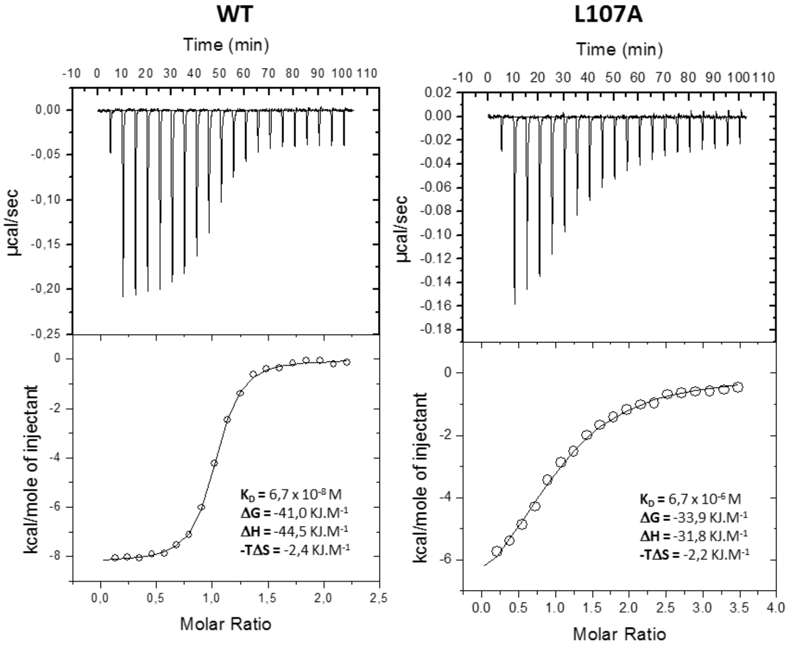


**Supplementary Figure 3** Representative ITC data for interactions between WT and L107A mutant NHSP90 with compounds **2** and **11**. The raw data are presented in the upper panels and the binding isotherms calculated from the total heat released for each injection are plotted against the molar ratio of injectant in the lower panels. The black lines show the calculated fitting curves applying a 1:1 interaction model.

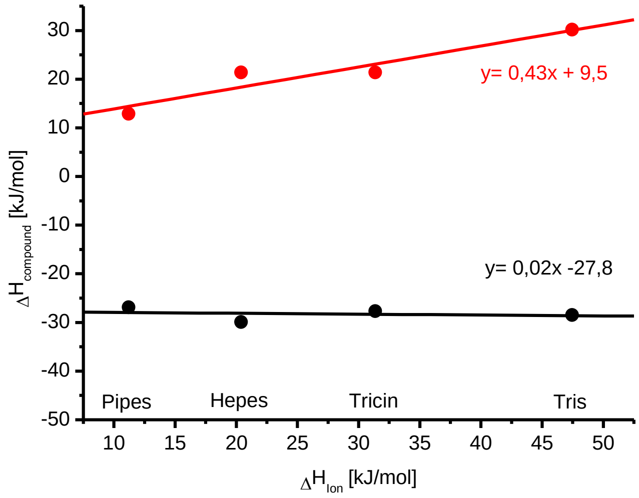


**Supplementary Figure 4** Potential protonation effects upon ligand binding. $\Delta H_{obs}$ is plotted against $\Delta H_{ion}$ for compounds **1** (in black) and **16** (in red) binding to N-HSP90. The points indicate the measured enthalpy values for each buffer system in kJ/mol. Linear fits to the data points are shown by continuous lines. Compound **1** does not experience changes in the protonation state upon binding whereas compound **16** displays a minor effect (fractional protonation of 0.43) of protonation.

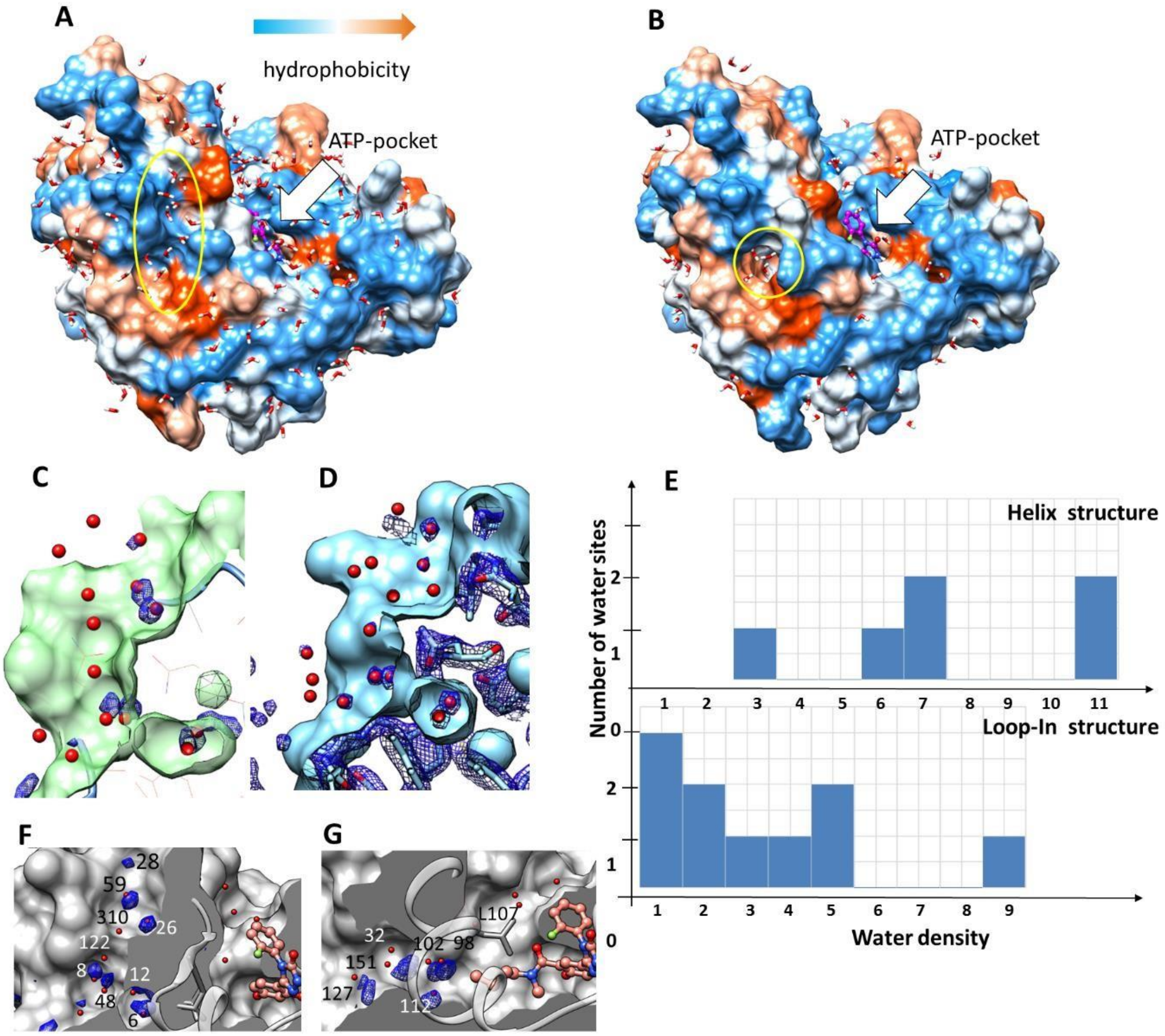


**Supplementary Figure 5** N-HSP90 showing the loop-in (**A**) and helical (**B**) conformations of the binding site (yellow ellipse; complexes with compounds **1** and **16**, respectively, were used as examples). The solvent accessible molecular surface of the protein is colored by hydrophobicity (rendered in Chimera[13]). (**C,D**) The water density distribution on the protein surface in the vicinity of the α-helix3 in a loop-in structure is shown as: (**C**) obtained from GIST[14] simulations at the isovalue of 2 (local water density with respect to the bulk) and (**D**) observed in crystal structure electron density distributions at the iso-value of 0. 4 (PDB:2BSM[15] is used for illustration). (**E**) Histogram of the maximum densities of stable water sites relative to the bulk, $\rho^{GIST}$, obtained by GIST analysis of MD trajectories (data are given in **Supplementary Table 6**), showing that the helical structure of the protein has more stable water sites around the α-helix3 than the loop-in one. However, the number of less stable water sites located on the protein surface (**B**) is larger for the loop-in conformation (**A**). (**F, G**) Positions of water molecules in the loop-in (PDB 5J64) and helical (PDB 5J9X) structures, respectively, are shown by red spheres, the blue mesh indicates the most stable water locations detected in MD simulations; numbers denote atom numbering in the corresponding PDB files.

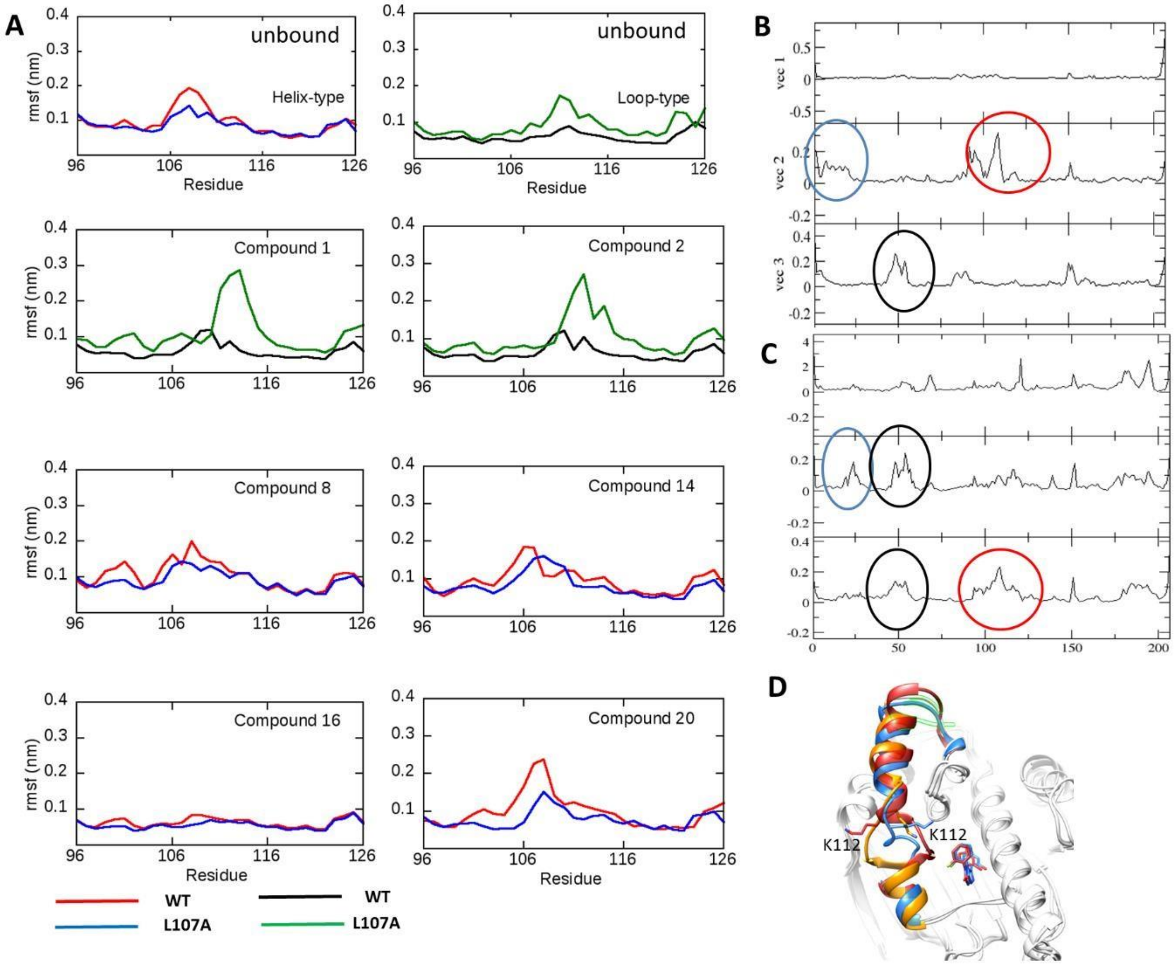


**Supplementary Figure 6** (**A**) RMSF of Cα atoms of α-helix3 (residues 96-126) of the WT N-HSP90 (red, black) and the L107A mutant (blue, green) along 1 μs MD simulation trajectories of the protein in apoform and complexed with six different ligands starting with the α-helix3 region in helical and loop-in conformations. These plots are complementary to the RMSD plots in **Fig. 5 B**; (**B, C**) First three PCA vectors of the protein backbone motion of N-HSP90 in helical (**B**) and loop-in (**C**) complexes (compounds **14** and **6**, respectively; residue numbering starts from GLU16). Motions of α-helix1, α -helix2, and α-helix3 are denoted by blue, black, and red circles, respectively. (**D**) Conformational changes of the α-helix3 region of N-HSP90 upon L107A mutation as observed in MD simulations of the complex. The crystal structure of the WT complex with compound **1** (with the α-helix3 region and ligand in red) was used as the starting conformation for MD simulation. The conformation of the mutant after 1μs of MD simulation is shown in blue. The conformation of the α-helix3 region in the crystal structure of the apo WT protein with a loop-out conformation (PDB ID; 1yes) is shown in orange for comparison. The side-chain of K112 in the MD simulations of the L107A mutant protein is rotated, adopting a position similar to that in the crystallographic loop-out conformation.

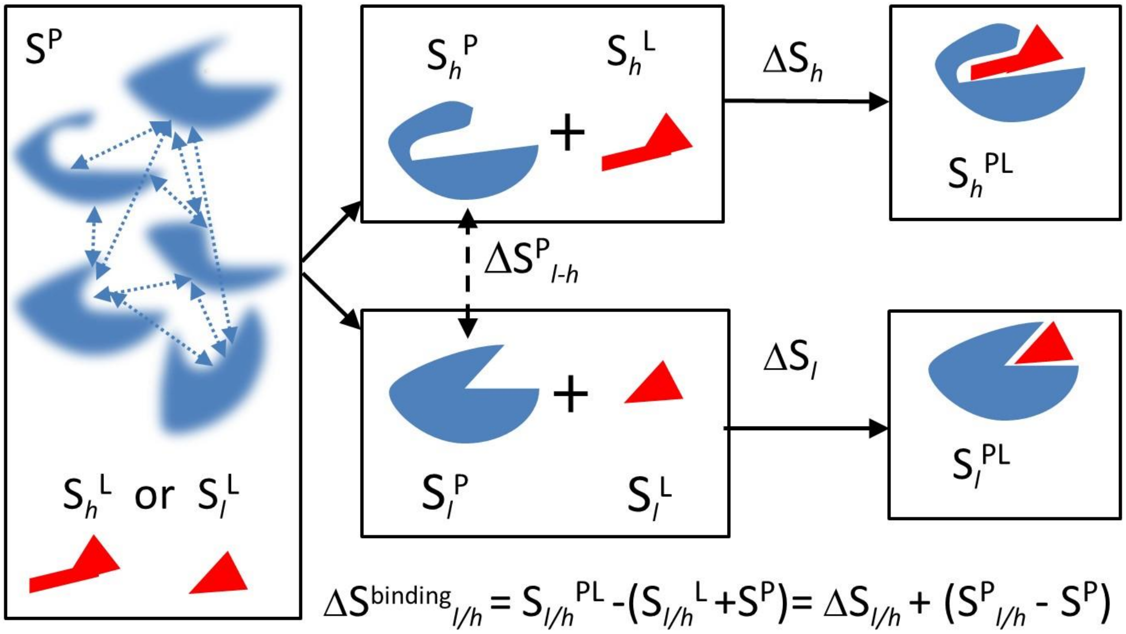


$$\Delta S^{binding}_{l/h} = S_{l/h}{}^{PL} - (S_{l/h}{}^{L} + S^P) = \Delta S_{l/h} + (S^P{}_{l/h} - S^P)$$

$$\Delta\, \Delta\, S^{binding}{}_{l\text{-}h} = \Delta S^{binding}{}_{l} - \Delta S^{binding}{}_{h} = (\Delta S_l - \Delta S_h\ ) + (S^P{}_l - S^P{}_h) = \Delta\Delta S_{l\text{-}h} + \Delta S^P{}_{l\text{-}h}$$

**Supplementary Figure 7** Illustration of the entropy decomposition considered for computing the relative conformational entropy contribution to the binding free energy of loop-in and helix-binding compounds, $S^{binding}{}_{l\text{-}h}$ . P denotes apo-protein, L denotes unbound ligand, and PL denotes a protein-ligand complex. *l* denotes loop-in and *h* denotes helical protein conformation, *l/h* denotes a value computed for either *l* or *h*, *l-h* denotes the difference between the loop-in and helix-conformations of the protein.

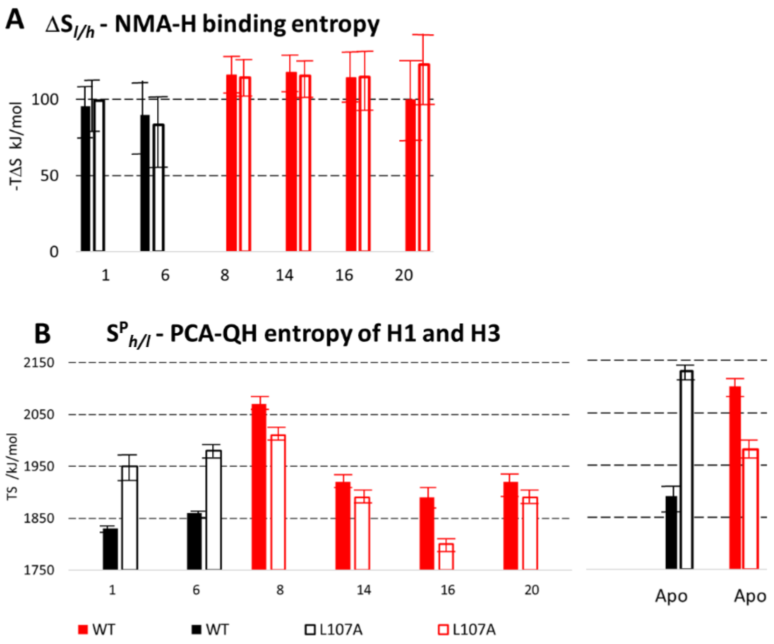


**Supplementary Figure 8** Computed entropic contributions to binding for compounds with available crystal structures: **1**,**6**,**8**,**14**, **16**, and **20.** Loop-in and helical structures are shown in black and red, respectively; filled and empty columns correspond to WT and L107A mutant structures, respectively. (**A**) Entropy change upon ligand binding arising from motion of both the protein and the ligand, computed assuming that the binding partners have the same conformation in apo- and holo-states using the NMA-H method. The mean and standard deviation values of the binding entropies computed for 50 frames extracted at equal time intervals from the first 10 ns of the MD trajectory for each complex are shown. (**B**) The entropy of the α-helix1 and α-helix3 segments in protein-ligand complexes and the apo-protein computed using the PCA-QH approximation. Snapshots extracted at 20 ps intervals over each MD trajectory were used to compute entropies. Four trajectory segments were analyzed (200-500 ns, 400-700 ns, 200-700 ns, and 100-800 ns) to evaluate the average entropy values and standard deviations shown for each system. See Sec. Methods for computational details.

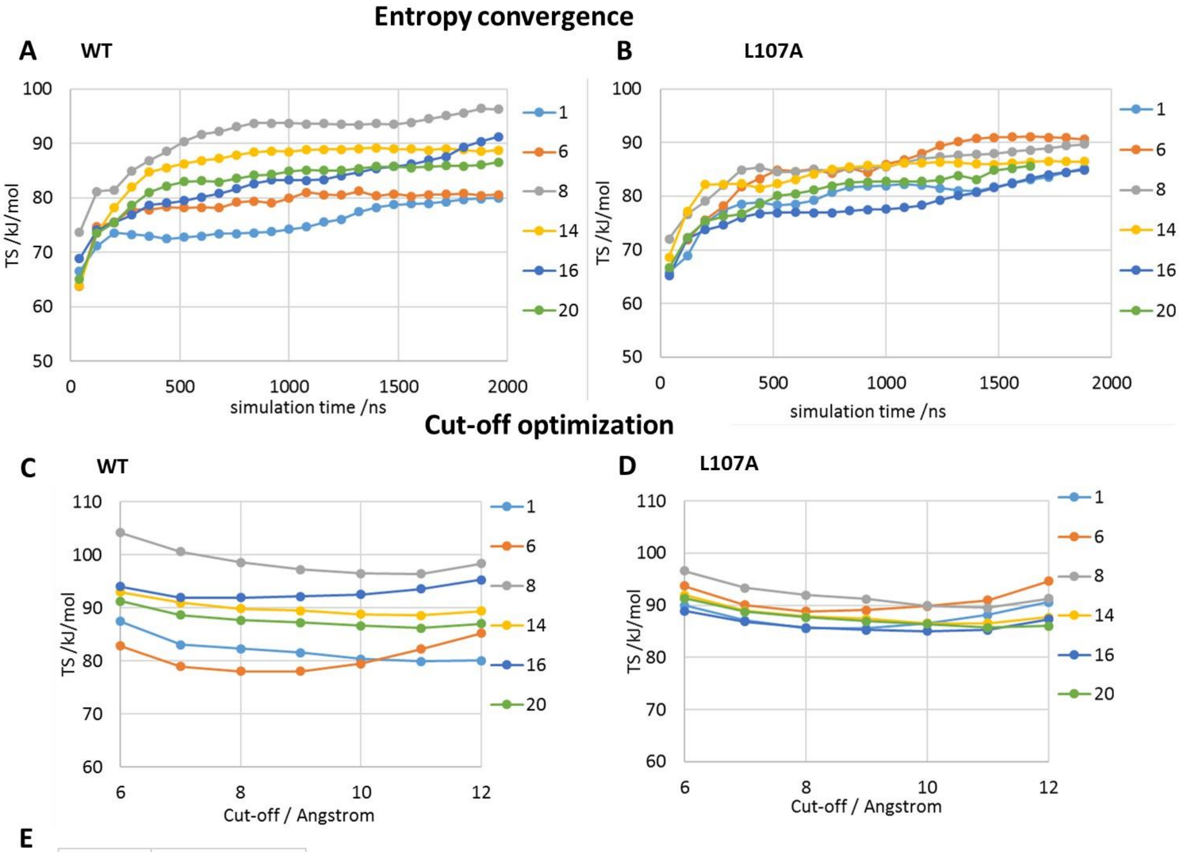


**E**

| | TS kJ/mol | |
|---|---|---|
| Compound | WT | L107A |
| 1 | 79.9 | 85.6 |
| 6 | 78.2 | 88.9 |
| 8 | 96.4 | 89.5 |
| 14 | 88.6 | 86.5 |
| 16 | 92 | 85 |
| 20 | 86.2 | 85.7 |

**Supplementary Figure 9** Details of **CC-MLA** entropy computation (simulation details are given in Section Methods): (**A-D**)- the energy term arising from the conformational entropy (the value at T=300K was used) is shown for different compounds as a function of the trajectory length included in simulations (a stride of 4 ps was employed, i.e. up to 500 000 snapshots from 2 μs MD simulations) (**A-B**) and as a function of the cutoff threshold. Entropy values are shown for WT **(A, C)** and for the L107A mutant **(B, D)** protein complexes; (**E**)– minimum values of the entropy terms from **Figs. C** and **D** for WT and L107A mutant HSP90, respectively.

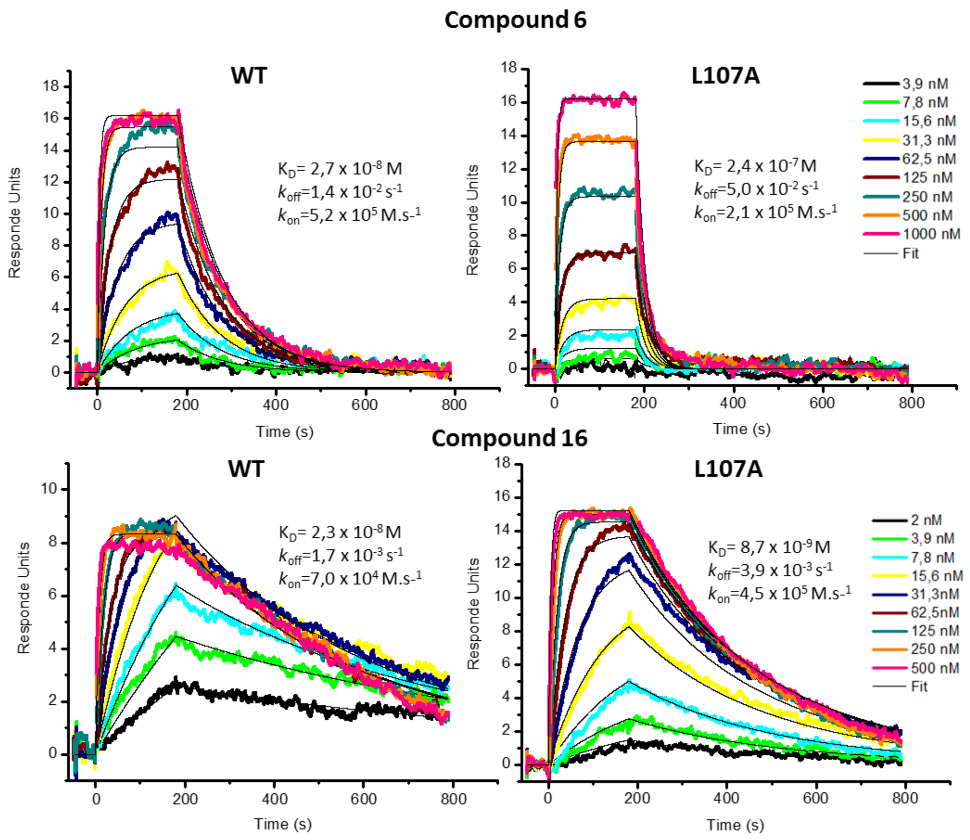


**Supplementary Figure 10** Representative sensograms obtained by SPR for interactions between WT and L107A mutant N-HSP90 with compounds **6** and **16.** Fitted model traces are shown by black lines.

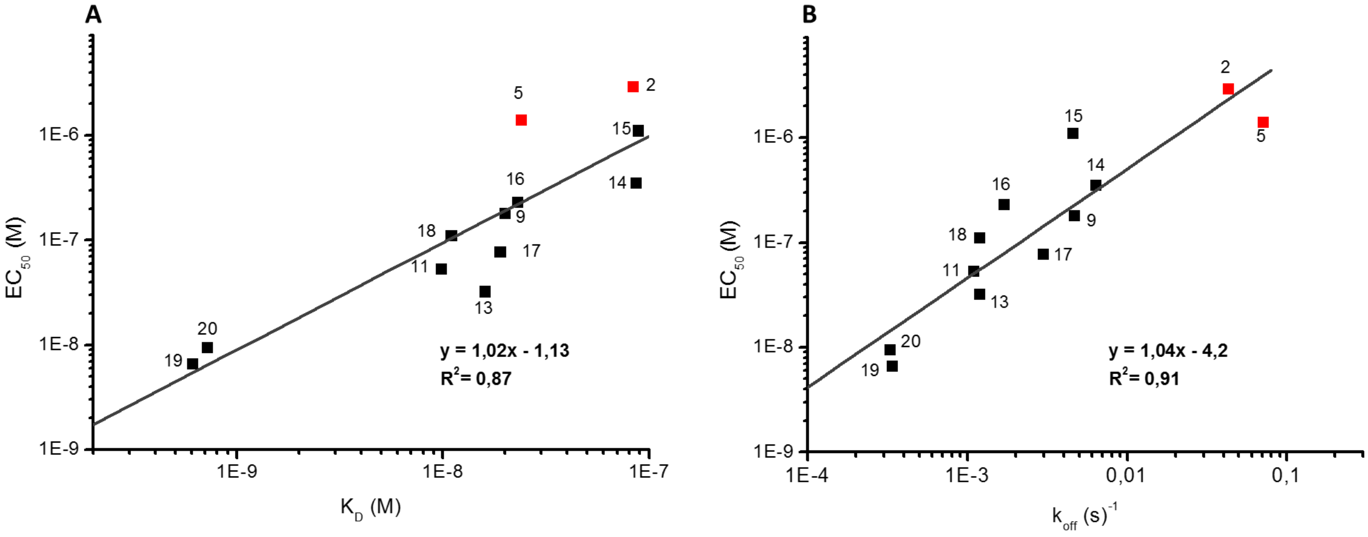


**Supplementary Figure 11** Correlation of *cellular* efficacy and kinetic constants: Logarithmic plot showing correlation of cellular efficacy determined by intracellular upregulation of HSP70 in A2780 cells (y-axis) with the equilibrium dissociation constant, $K_D$ (**A**) and the dissociation rate constant, $k_{off}$ (x-axis) (**B**). Compounds assigned as loop-binders are colored black and compounds assigned as helix-binders are colored red. The grey line is the linear regression, with $R^2$ representing the coefficient of determination. A significant linear relationship between efficacy and $k_{off}$ strongly supports the idea that slow drug–complex dissociation is a critical molecular determinant of sustained pharmacological activity *in vivo*.

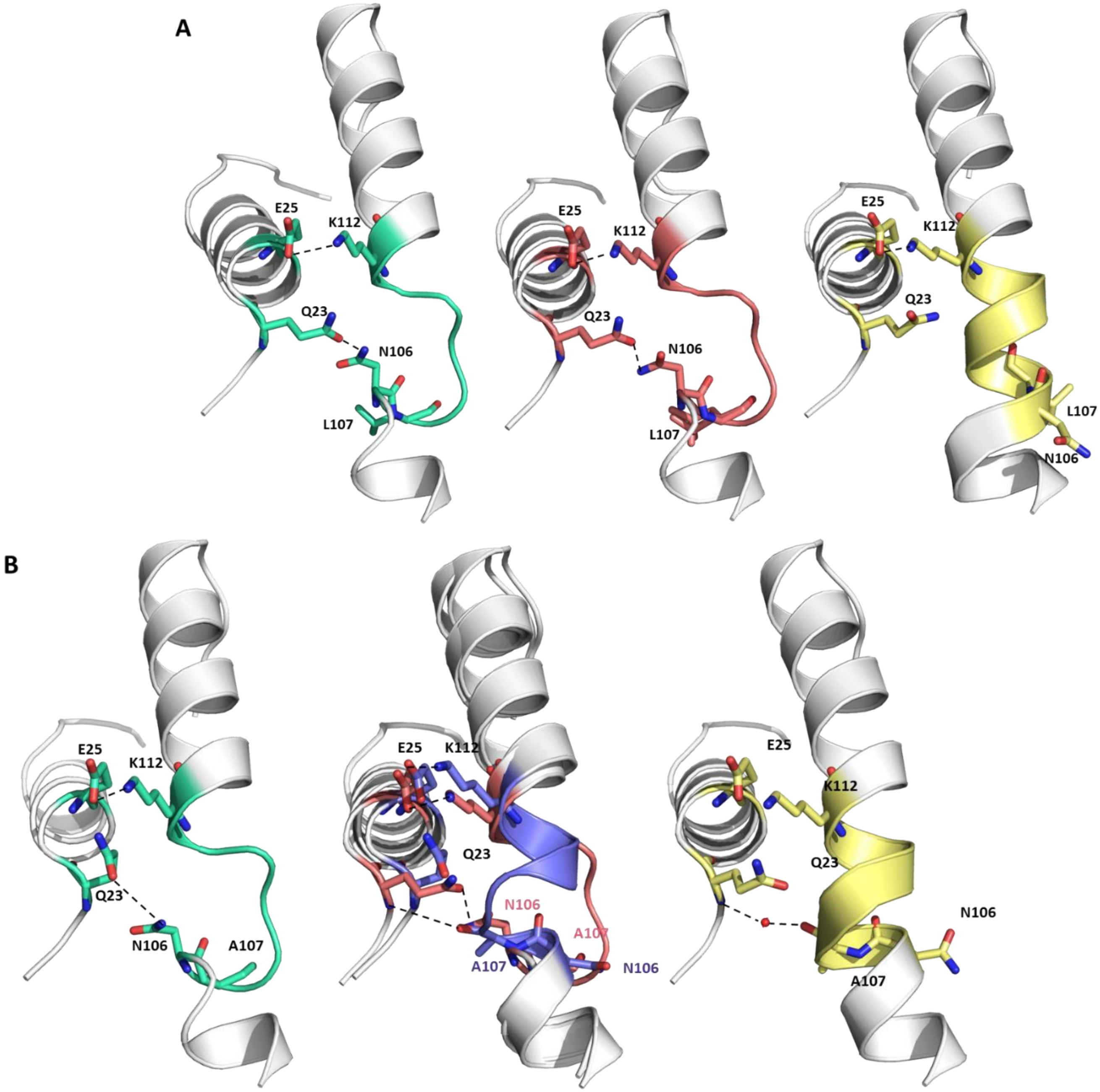


**Supplementary Figure 12** Comparison of $\alpha$-helix1 and $\alpha$-helix3 from crystal structures of N-HSP90 in free state (loop-in conformation, relevant residues colored in cyan, left) and in complex with compounds **1** (loop conformation, colored in salmon and alternate conformation colored in violet, middle) and **16** (helical conformation, colored in yellow, right) in WT (**A**) and L107A mutant (**B**) N-HSP90. (**A**) In loop-in structures, the $\alpha$-helix3 region forms hydrogen bonds (represented by the black dashed lines) with $\alpha$-helix1 between E25-K112 and Q23-N106. In helical structures (exemplified by the crystal structure of NHSP90 in complex with compound **16**), the Q23-N106 interaction is not possible due to the binding site rearrangement. N106 is rotated away by about 180$^o$. (**B**) The crystal structure of the unbound L107A mutant preserves the same interactions between $\alpha$-helix3 and $\alpha$-helix1 as for the WT apo-protein with A107 occupying the same position as L107 in the WT. The complexes with the loop binding compounds

(as exemplified for the complex of the L107A mutant with compound **1**) exist in two alternate conformations after L107A mutation: loop-in (colored in salmon) and loop-out (colored in blue) conformations. The A107 side-chain of the loop-in conformation resembles that of the unbound L107A mutant. However, the A107 of the loop-out conformation does not occupy the same position as L107 in the WT, but is rotated towards $\alpha$-helix1. The backbone carbonyl of A107 forms a hydrogen bond with the amide backbone of Q23. In helical conformations of the L107A mutant, the A107 side-chain is also rotated towards $\alpha$-helix1 as for loop-out conformation. The backbone carbonyl of A107 forms a hydrogen bond with the amide backbone of Q23 directly or through a water bridge (as exemplified for the complex of the L107A mutant with compound **16**).

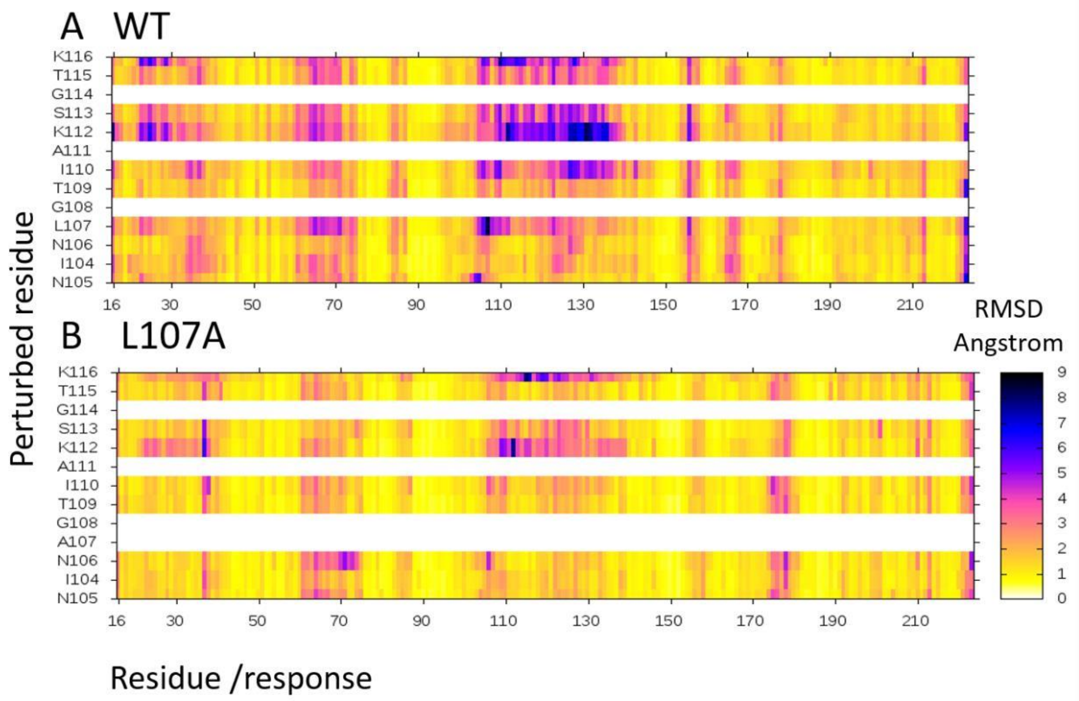


**Supplementary Figure 13** L-RIP perturbation MD simulations (see Methods) of unbound N-HSP90 starting in the helical conformation (**A**)-WT and (**B**)-L107A mutant. The perturbed residues (y-axis) are in the $\alpha$–helix3 region; the response is given by the structural deviation along the protein sequence (x-axis). The RMSD is colored increasing from yellow to black.

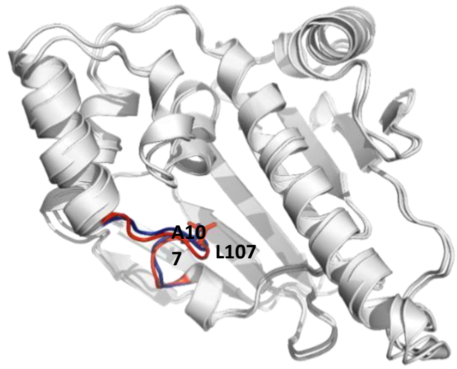


**Supplementary Figure 14** Overlay of crystal structures of N-HSP90 WT and L107A mutant in the apo state. The position of L107 in WT is occupied by A107 in the L107A mutant. L107 and A107 are represented by sticks. The loop region is colored red in the WT and blue in the L107A mutant.

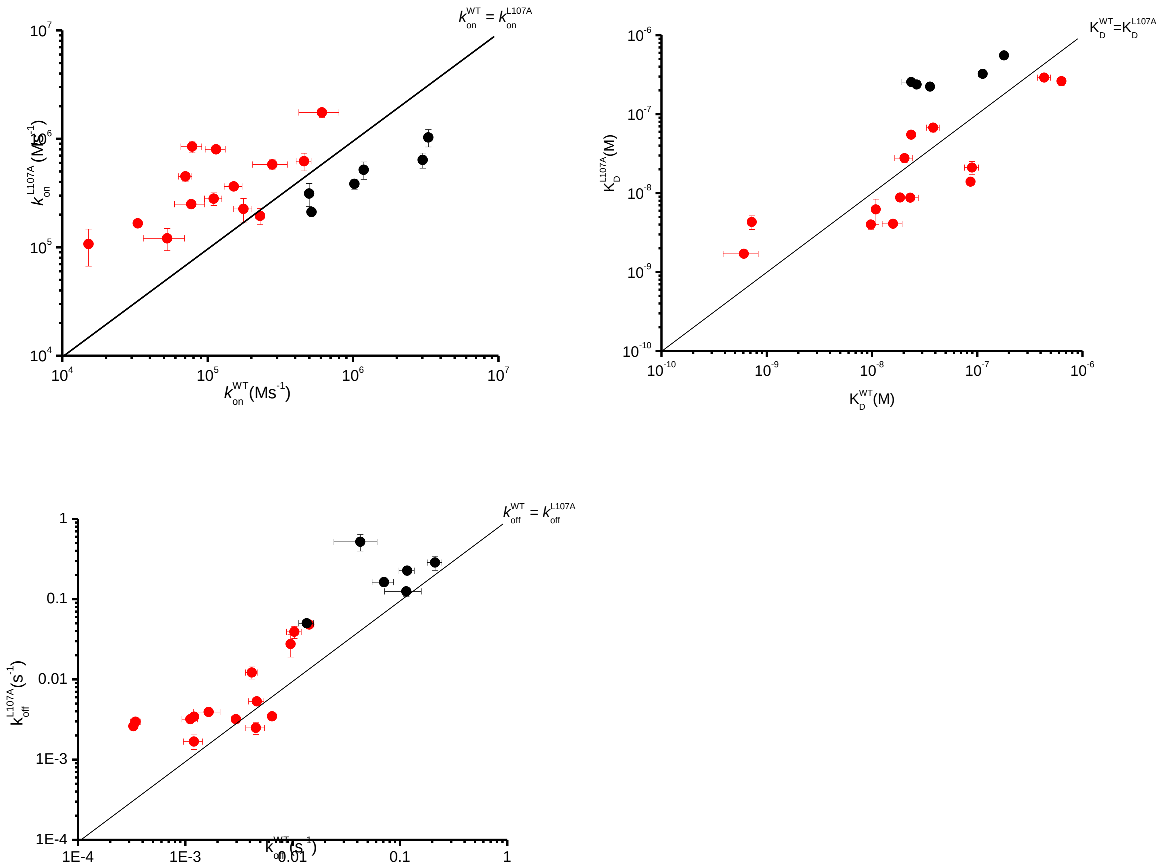


**Supplementary Figure 15** Plot of association ($k_{on}$) rate constant, dissociation ($k_{off}$) rate constant, and equilibrium dissociation constant ($K_D$) for compounds **1-20** for the L107A mutant versus WT N-HSP90. Compounds assigned as loop-binders are colored black and compounds assigned as helix-binders are colored red.

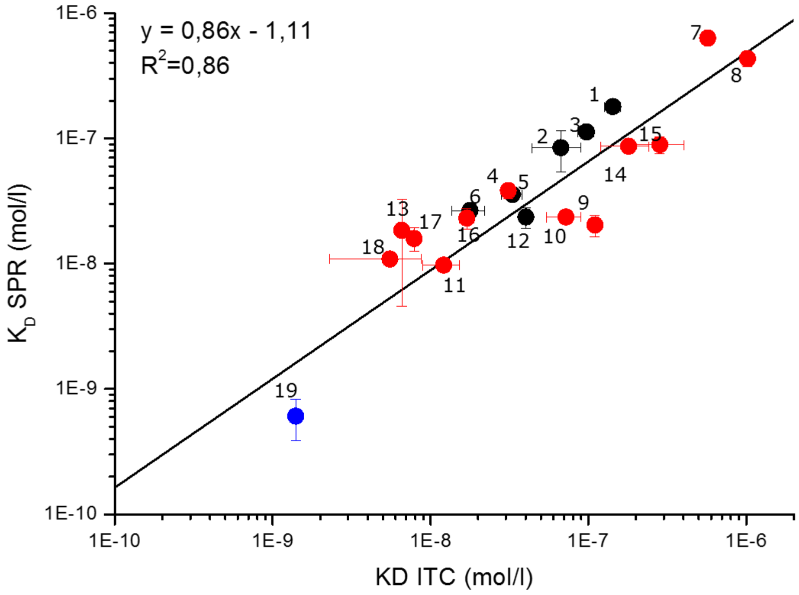


**Supplementary Figure 16** Equilibrium dissociation constants, $K_D$, obtained from SPR (y axis) plotted against those obtained from ITC (x axis) experiments for compounds **1-19**. Loop binders are shown in black, helix binders in red. Compound **19** (helix binder) is marked in blue, as its $K_D$ values are close to the resolution limit of ITC and SPR assays (~1 nM). The linear fit ($R^2$=0.86, with an average fold difference of 2) to all data points is shown by the black line. The only outlier is compound 9 with a maximum fold difference of 6.

# Supplementary Tables

**Supplementary Table 1** Data collection and refinement statistics

| | ***Apo*** | ***1*** | ***6*** | ***8*** | ***14*** |
|---|---|---|---|---|---|
| ***Data collection*** | | | | | |
| *Space group* | *I222* | *I222* | I222 | I222 | I222 |
| *Cell dimensions* | | | | | |
| *a, b, c (Å)* | 67.77 92.40 103.53 | 65.63 88.89 99.65 | 66.36 89.19 99.86 | 67.08 90.24 98.09 | 70.42 88.99 96.93 |
| *α, β, γ (°)* | 90.00, 90.00, 90.00 | 90.00, 90.00, 90.00 | 90.00, 90.00, 90.00 | 90.00, 90.00, 90.00 | 90.00, 90.00, 90.00 |
| *Resolution (Å)* | 46.20-1.59 (1.69-1.59) | 44.45-1.38 (1.46-1.38) | 44.59-1.22 (1.29-1.22) | 36.26-1.87 (1.98-1.87) | 44.50-1.70 (1.80-1.70) |
| *Nr. observations* | 288614 (44296) | 391091 (60200) | 552358 (79075) | 165189 (26689) | 209380 (31916) |
| *Unique reflections* | 43963 (7025) | 60097 (9323) | 86757 (13285) | 24957 (3945) | 33922 (5321) |
| *Redundancy* | 6.56 | 6.50 | 6.36 | 6.61 | 26.60 |
| *Completeness (%)* | 99.9 (99.60) | 99.1 (96.0) | 97.6 (93.1) | 99.9 (99.4) | 99.6 (98.1) |
| $R_{meas}$ *(%)* | 4.7 (57.6) | 5.1 (86.2) | 5.6 (142.5) | 5.7 (205.7) | 6.8 (173.8) |
| $<I/\sigma(I)>$ | 23.71 (3.05) | 17.7 (1.87) | 14.48 (0.95) | 14.31 (0.77) | 16.10 (0.99) |
| *CC 1/2* | 1.0 (0.85) | 1.0 (0.79) | 1.0 (0.45) | 1.0 (0.45) | 1.00 (0.44) |
| ***Refinement*** | | | | | |
| *Resolution (Å)* | 46.20-1.59 (1.63-1.59) | 44.45-1.38 (1.42-1.38) | 44.59-1.22 (1.25-1.22) | 36.26-1.87 (1.95-1.87) | 44.50-1.70 (1.75-1.70) |
| $R_{work}$ *(%)* | 19.7 | 18.5 | 19.9 | 20.6 | 19.9 |
| $R_{free}$ *(%)* | 20.3 | 20.0 | 22.9 | 21.7 | 22.9 |
| *Model composition* | | | | | |

| | | | | | |
|---|---|---|---|---|---|
| <u>*and completeness*</u> | | | | | |
| *Protein* | 1668 | 1636 | 1693 | 1636 | 1664 |
| *Ligand* | - | 21 | 44 | 31 | 29 |
| *solvent* | 365 | 315 | 306 | 91 | 212 |
| *Average B factor all atoms (Å$^2$)* | 25.6 | 26.62 | 22.0 | 64.0 | 37.94 |
| <u>*Model validation*</u> | | | | | |
| *% Ramachandran outliers* | 0 | 0 | 0 | 0 | 0 |
| *% Ramachandran favored* | 100 | 97 | 98 | 97 | 96 |
| *% Rotamer outliers* | 0 | 0 | 0 | 0 | 0 |
| *PDB code* | 5J2V | 5J64 | 5J2X | 5J86 | 5J27 |

| | ***16*** | ***18*** | ***20*** | ***Apo-L107A*** | ***1-L107A*** |
|---|---|---|---|---|---|
| ***Data collection*** | | | | | |
| *Space group* | *I222* | I222 | I222 | *I222* | *C121* |
| *Cell dimensions* | | | | | |
| *a, b, c (Å)* | 67.89 91.33 98.96 | 67.08 90.24 98.09 | 69.92 88.66 97.88 | 64.78 86.40 99.63 | 130.24 64.95 88.11 |
| *α, β, γ (°)* | 90.00, 90.00, 90.00 | 90.00, 90.00, 90.00 | 90.00, 90.00, 90.00 | 90.00, 90.00, 90.00 | 90.00 130.34 90.00 |
| *Resolution (Å)* | 45.67-1.80 (1.84-1.80) | 36.26-1.87 (1.98-1.87) | 22.46–1.76 (1.87-1.76) | 43.16-1.17 (1.25-1.17) | 44.02-1.75 (1.85-1.75) |
| *Nr. observations* | 168191 (11314) | 165189 (26689) | 196824 (29461) | 689044 (99484) | 183398 (25506) |
| *Unique reflections* | 28122 (1705) | 24957 (3945) | 30501 (4830) | 111027 (16837) | 56286 (8415) |
| *Redundancy* | 5.98 | 6.61 | 6.45 | 6.20 | 3.25 |
| *Completeness (%)* | 97.5 (100.0) | 99.9 (99.4) | *99.8 (99.0)* | 98.1 (92.2) | 97.4 (91.3) |
| *$R_{meas}$ (%)* | 4.44 (131.7) | 5.7 (205.7) | 6.7 (208.8) | 5.2 (90.1) | 8.2 (134.8) |
| *$<I/\sigma(I)>$* | 21.5 (1.3) | 14.31 (0.77) | 18.56 (0.90) | 13.83 (1.53) | 8.42 (0.67) |
| *CC 1/2* | 1.0 (0.62) | 1.0 (0.45) | 0.99 (0.43) | 1.0 (0.73) | 0.99 (0.52) |
| ***Refinement*** | | | | | |
| *Resolution (Å)* | 36.63-1.80 (1.87-1.80) | 36.26-1.87 (1.95-1.87) | 22.46–1.76 (1.82-1.76) | 43.16-1.17 (1.20-1.17) | 44.02-1.75 (1.79-1.75) |
| *$R_{work}$ (%)* | 18.58 | 20.6 | 19.8 | 18.0 | 21.9 |
| *$R_{free}$ (%)* | 23.44 | 22.9 | 19.1 | 24.7 | 22.9 |
| *Model composition and completeness* | | | | | |
| *Protein* | 1636 | 1636 | 1632 | 1632 | 3256 |
| *Ligand* | 29 | 31 | 31 | - | 42 |

| | | | | | |
|---|---|---|---|---|---|
| *solvent* | 169 | 91 | 251 | 377 | 445 |
| *Average B factor all atoms (Å$^2$)* | 49.0 | 64.0 | 40.0 | 21.29 | 34.65 |
| <u>*Model validation*</u> | | | | | |
| *% Ramachandran outliers* | 1 | 0 | 0 | 0 | 0 |
| *% Ramachandran favored* | 96 | 97 | 97 | 96 | 96 |
| *% Rotamer outliers* | 2 | 0 | 0 | 0 | 0 |
| *PDB code* | 5J9X | 5J86 | 5J20 | 5J80 | 5J8U |

| | *6-L107A* | *14-L107A* | *16-L107A* | *20-L107A* |
|---|---|---|---|---|
| ***Data collection*** | | | | |
| *Space group* | *C121* | I222 | I222 | I222 |
| *Cell dimensions* | | | | |
| *a, b, c (Å)* | 129.79 65.19 88.01 | 67.62 88.41 98.75 | 67.21 90.01 99.52 | 67.17 89.69 98.48 |
| *α, β, γ (°)* | 90.00 130.11 90.00 | 90.00, 90.00, 90.00 | 90.00, 90.00, 90.00 | 90.00, 90.00, 90.00 |
| *Resolution (Å)* | 43.96-1.90 (2.02-1.90) | 21.89-1.90 (2.00-1.89) | 22.48-1.75 (1.86-1.75) | 22.69-1.64 (1.73-1.64) |
| *Nr. observations* | 135825 (16159) | 148589 (20274) | 195610 (30558) | 181720 (26641) |
| *Unique reflections* | 42717 (6227) | 23786 (3474) | 30736 (4850) | 36998 (5588) |
| *Redundancy* | 3.17 | 6.24 | 6.34 | 4.91 |
| *Completeness (%)* | 96.3 (87.2) | 98.3 (90.5) | 99.8 (99.2) | 98.7 (93.5) |
| $R_{meas}$*(%)* | 10.3 (88.8) | 8.5 (109.7) | 5.8 (177.3) | 5.5 (110.8) |
| $\langle I/\sigma(I)\rangle$ | 8.04 (1.07) | 16.67 (1.52) | 20.75 (0.93) | 14.00 (1.11) |
| *CC 1/2* | 1.0 (0.50) | 1.0 (0.65) | 1.0 (0.51) | 1.0 (0.62) |
| ***Refinement*** | | | | |
| *Resolution (Å)* | 43.96-1.90 (1.95-1.90) | 21.89-1.90 (1.98-1.90) | 22.48-1.75 (1.81-1.75) | 22.69-1.64 (1.69-1.64) |
| $R_{work}$ *(%)* | 18.9 | 20.8 | 20.8 | 20.3 |
| $R_{free}$ *(%)* | 21.3 | 23.6 | 22.5 | 23.6 |
| *Model composition and completeness* | | | | |
| *Protein* | 3283 | 1637 | 1629 | 1655 |
| *Ligand* | 44 | 29 | 29 | 31 |
| *solvent* | 366 | 245 | 203 | 238 |

| | | | | |
|---|---|---|---|---|
| *Average B factor all atoms (Å$^2$)* | 34.89 | 39.10 | 40.4 | 35.17 |
| *<u>Model validation</u>* | | | | |
| *% Ramachandran outliers* | 0 | 0 | 0 | 0 |
| *% Ramachandran favored* | 97 | 98 | 99 | 98 |
| *% Rotamer outliers* | 1 | 0 | 1 | 0 |
| *PDB code* | 5J8M | 5J6N | 5J6L | 5J6M |

**Supplementary Table 2** Structures and thermodynamic parameters of inhibitors bound to WT and L107A N-HSP90. The data for the WT represent the average of two titrations. The $K_D$ fold difference was calculated as the ratio of the WT to the L107A binding constants. □□H, □□-T□S) and □□G values were calculated by subtracting the respective WT thermodynamic parameters from those of the L107A mutant.

| | | | WT | | | | L107A | | | | Fold difference $K_D$ | ΔΔH | Δ(-TΔS) | ΔΔG |
|---|---|---|---|---|---|---|---|---|---|---|---|---|---|---|
| Compound ID | R[1] | R[2] | KD. M | ΔH. kJ.M$^{-1}$ | -TΔS. kJ.M$^{-1}$ | ΔG. kJ.M$^{-1}$ | $K_D$. M | ΔH. kJ.M$^{-1}$ | -TΔS. kJ.M$^{-1}$ | ΔG. kJ.M$^{-1}$ | | | | |
| 1 | H | F | $1.4 \times 10^{-7} \pm 1.7 \times 10^{-8}$ | -29.1±1.1 | -10.1±1.3 | -39.1±0.3 | $3.4 \times 10^{-7}$ | -29.7 | -7.5 | -37.1 | 0.42 | -0.52 | 2.56 | 2.00 |
| 2 | I | F | $6.0 \times 10^{-8} \pm 5.7 \times 10^{-9}$ | -37.9±4.3 | -3.5±4.5 | -41.2±0.2 | $1.2 \times 10^{-6}$ | -31.8 | -2.2 | -33.9 | 0.05 | 6.13 | 1.27 | 7.37 |
| 3 | H | Me | $9.7 \times 10^{-8} \pm 1.1 \times 10^{-8}$ | -35.5±0.6 | -4.8±0.9 | -40.0±0.3 | $8.4 \times 10^{-7}$ | -35.9 | 1.1 | -34.7 | 0.12 | -0.42 | 5.82 | 5.36 |
| 4 | H | Cl | $3.3 \times 10^{-8} \pm 5.0 \times 10^{-9}$ | -42.3±0.5 | 1.05±0.9 | -42.7±0.4 | $3.8 \times 10^{-7}$ | -35.9 | -0.9 | -36.7 | 0.09 | 6.42 | -1.99 | 6.05 |
| 5 | H | Et | $4.0 \times 10^{-8} \pm 3.6 \times 10^{-9}$ | -48.7±0.2 | 6.3±0.1 | -42.2±0.2 | $2.6 \times 10^{-7}$ | -33.1 | -4.7 | -37.6 | 0.16 | 15.67 | -11.01 | 4.60 |
| 6 | Br | F | $1.8 \times 10^{-8} \pm 4.2 \times 10^{-9}$ | -27.2±0.2 | -17.3±0.4 | -44.2±0.6 | $1.6 \times 10^{-7}$ | -20.8 | -18.2 | -38.8 | 0.11 | 6.38 | -0.88 | 5.41 |
| 7*[1] | 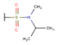 | Cl | $6.6 \times 10^{-7} \pm 1.3 \times 10^{-7}$ | 26.7± 1.1 | -62.1 ± 1.6 | -35.3 ± 0.5 | - | - | - | - | | | | |
| 8 | 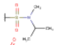 | F | $1.0 \times 10^{-6} \pm 4.0 \times 10^{-8}$ | 31.0±2.3 | -65.3±2.2 | -34.2±0.1 | $2.9 \times 10^{-7}$ | -21.1 | -10.6 | -31.6 | 0.35 | -52.09 | 54.68 | 2.63 |
| 9 | 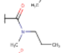 | Me | $1.3 \times 10^{-7} \pm 2.8 \times 10^{-8}$ | 24.1± 0.0 | -63.6 ± 0.5 | -39.3± 0.5 | $3.0 \times 10^{-8}$ | 7.0 | -50.1 | -42.9 | 4.33 | -17.13 | 13.52 | -3.60 |
| 10 | 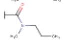 | F | $7.2 \times 10^{-8} \pm 1.8 \times 10^{-8}$ | 26.1±1.7 | -67.0±1.1 | -40.8±0.6 | $2.8 \times 10^{-8}$ | -2.9 | -40.3 | -43.1 | 2.54 | -28.94 | 26.66 | -2.27 |
| 11 | 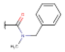 | Me | $1.2 \times 10^{-8} \pm 3.2 \times 10^{-9}$ | 13.7±0.8 | -59.1±1.4 | -45.2±0.7 | $1.6 \times 10^{-8}$ | -16.3 | -28.5 | -44.6 | 0.76 | -30.02 | 30.60 | 0.65 |
| 12 | 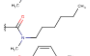 | Me | $4.9 \times 10^{-8} \pm 2.6 \times 10^{-8}$ | 8.8±1.4 | -50.8 ± 0.0 | -41.9 ± 1.4 | $3.4 \times 10^{-7}$ | -9.1 | -27.9 | -36.9 | 0.15 | -17.93 | 22.90 | 4.96 |
| 13*[1] | 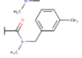 | Me | $6.0 \times 10^{-9} \pm 2.7 \times 10^{-9}$ | 6.5±0.5 | -53.7± 1.6 | -47.1 ± 1.2 | - | - | - | - | | | | |
| 14 | 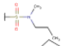 | F | $1.8 \times 10^{-7} \pm 6.1 \times 10^{-8}$ | 19.6±2.5 | -58.3±1.6 | -38.6±0.8 | $1.7 \times 10^{-8}$ | -35.1 | -9.5 | -44.4 | 10.83 | -54.68 | 48.82 | -5.84 |
| 15 | 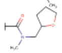 | Me | $2.8 \times 10^{-7} \pm 1.2 \times 10^{-7}$ | 15.3±3.2 | -52.8±2.1 | -37.5±1.1 | $1.5 \times 10^{-7}$ | -33.3 | -5.7 | -38.9 | 1.88 | -48.60 | 47.09 | -1.45 |
| 16 | 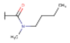 | F | $1.7 \times 10^{-8} \pm 1.8 \times 10^{-9}$ | 21.4±0.1 | -67.0±3.4 | -45.3±3.4 | $1.3 \times 10^{-8}$ | -14.8 | -30.5 | -45.1 | 1.34 | -36.27 | 36.48 | 0.21 |
| 17 | 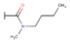 | - | $9.5 \times 10^{-9} \pm 4.1 \times 10^{-9}$ | 15.5 ± 0.3 | -61.6± 1.4 | -45.9 ± 1.1 | $4.6 \times 10^{-9}$ | -12.9 | -34.9 | -47.6 | 2.08 | -28.35 | 26.66 | -1.70 |
| 18 | 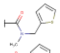 | Me | $5.5 \times 10^{-9} \pm 3.2 \times 10^{-9}$ | 11.4±1.7 | -59.0±0.2 | -47.3±1.5 | $1.4 \times 10^{-8}$ | -36.2 | -8.9 | -44.9 | 0.41 | -47.63 | 50.02 | 2.40 |
| 19 | 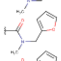 | Me | $1.6 \times 10^{-9} \pm 2.2 \times 10^{-10}$ | -8.7 ± 0.7 | -41.8± 0.4 | -50.5± | $4.2 \times 10^{-9}$ | -16.2 | -31.8 | -47.9 | 0.37 | -7.50 | 10.00 | 2.37 |
| 20*[2] | 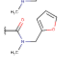 | F | $7.2 \times 10^{-10}$ | 0.0 | -52.2 | -52.2± | $2.7 \times 10^{-9}$ | -26.4 | -22.9 | -49.1 | 0.27 | -26.38 | 29.30 | 3.13 |

*[1] Accurate determination of binding affinity and enthalpy for the L107A mutant was not possible due to low reproducibility.

*[2] The heat of binding is near zero for WT. Entropy was estimated based on the binding affinity measured by SPR.

**Supplementary Table 3** Structure and kinetic parameters of inhibitors bound to WT and L107A N-HSP90. The data represent the average of 3-4 individual measurements. $K_D$, $k_{on}$ and $k_{off}$ fold differences were calculated as the ratio of the respective WT constant to the L107A constant.

| Compound ID | R¹ | R² | WT | | | L107A | | | Fold increase $K_D$ | Fold decrease $k_{on}$ | Fold increas $k_{off}$ |
|---|---|---|---|---|---|---|---|---|---|---|---|
| | | | $K_D$, M | $k_{on}$, $M^{-1}s^{-1}$ | $k_{off}$, $s^{-1}$ | $K_D$, M | $k_{on}$, $M^{-1}s^{-1}$ | $k_{off}$, $s^{-1}$ | | | |
| 1 | H | F | $1.8 \times 10^{-7} \pm 1.2 \times 10^{-8}$ | $1.2 \times 10^{6} \pm 2.1 \times 10^{5}$ | $2.1 \times 10^{-1} \pm 3.3 \times 10^{-2}$ | $5.5 \times 10^{-7} \pm 2.7 \times 10^{-8}$ | $5.2 \times 10^{5} \pm 9.4 \times 10^{4}$ | $2.9 \times 10^{-1} \pm 5.7 \times 10^{-2}$ | 0.3 | 2.3 | 0.7 |
| 2 | I | F | $8.4 \times 10^{-8} \pm 3.1 \times 10^{-8}$ | $5.0 \times 10^{5} \pm 4.9 \times 10^{4}$ | $4.3 \times 10^{-2} \pm 1.9 \times 10^{-2}$ | $1.7 \times 10^{-6} \pm 1.1 \times 10^{-7}$ | $3.1 \times 10^{5} \pm 7.5 \times 10^{4}$ | $5.2 \times 10^{-1} \pm 1.2 \times 10^{-1}$ | 0.1 | 1.6 | 0.1 |
| 3 | H | Me | $1.1 \times 10^{-7} \pm 1.0 \times 10^{-8}$ | $1.0 \times 10^{6} \pm 4.1 \times 10^{5}$ | $1.1 \times 10^{-1} \pm 4.3 \times 10^{-2}$ | $3.2 \times 10^{-7} \pm 1.1 \times 10^{-8}$ | $3.8 \times 10^{5} \pm 4.1 \times 10^{4}$ | $1.3 \times 10^{-1} \pm 1.6 \times 10^{-2}$ | 0.3 | 2.7 | 0.9 |
| 4 | H | Cl | $3.6 \times 10^{-8} \pm 2.0 \times 10^{-9}$ | $3.3 \times 10^{6} \pm 7.2 \times 10^{5}$ | $1.2 \times 10^{-1} \pm 2.0 \times 10^{-2}$ | $2.2 \times 10^{-7} \pm 2.0 \times 10^{-8}$ | $1.0 \times 10^{6} \pm 1.9 \times 10^{5}$ | $2.3 \times 10^{-1} \pm 2.7 \times 10^{-2}$ | 0.2 | 3.2 | 0.5 |
| 5 | H | Et | $2.4 \times 10^{-8} \pm 4.3 \times 10^{-9}$ | $3.0 \times 10^{6} \pm 3.5 \times 10^{5}$ | $7.1 \times 10^{-2} \pm 1.6 \times 10^{-2}$ | $2.6 \times 10^{-7} \pm 9.5 \times 10^{-9}$ | $6.4 \times 10^{5} \pm 1.0 \times 10^{5}$ | $1.6 \times 10^{-1} \pm 2.1 \times 10^{-2}$ | 0.1 | 4.7 | 0.4 |
| 6 | Br | F | $2.7 \times 10^{-8} \pm 2.6 \times 10^{-9}$ | $5.2 \times 10^{5} \pm 1.3 \times 10^{5}$ | $1.4 \times 10^{-2} \pm 2.2 \times 10^{-3}$ | $2.4 \times 10^{-7} \pm 7.1 \times 10^{-9}$ | $2.1 \times 10^{5} \pm 1.5 \times 10^{4}$ | $5.0 \times 10^{-2} \pm 2.4 \times 10^{-3}$ | 0.1 | 2.5 | 0.3 |
| 7 | 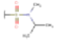 | Cl | $6.3 \times 10^{-7} \pm 2.9 \times 10^{-8}$ | $1.5 \times 10^{4} \pm 1.6 \times 10^{2}$ | $9.6 \times 10^{-3} \pm 5.4 \times 10^{-4}$ | $2.6 \times 10^{-7} \pm 3.1 \times 10^{-8}$ | $1.1 \times 10^{5} \pm 4.0 \times 10^{4}$ | $2.8 \times 10^{-2} \pm 8.6 \times 10^{-3}$ | 2.4 | 0.1 | 0.3 |
| 8 | 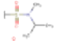 | F | $4.3 \times 10^{-7} \pm 6.1 \times 10^{-8}$ | $3.3 \times 10^{4} \pm 1.3 \times 10^{3}$ | $1.4 \times 10^{-2} \pm 1.5 \times 10^{-3}$ | $2.9 \times 10^{-7} \pm 2.4 \times 10^{-8}$ | $1.7 \times 10^{5} \pm 6.6 \times 10^{3}$ | $4.8 \times 10^{-2} \pm 3.0 \times 10^{-3}$ | 1.5 | 0.2 | 0.3 |
| 9 | 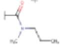 | Me | $2.0 \times 10^{-8} \pm 4.0 \times 10^{-9}$ | $2.3 \times 10^{5} \pm 1.5 \times 10^{4}$ | $4.7 \times 10^{-3} \pm 8.0 \times 10^{-4}$ | $2.8 \times 10^{-8} \pm 3.3 \times 10^{-9}$ | $2.0 \times 10^{5} \pm 3.4 \times 10^{4}$ | $5.3 \times 10^{-3} \pm 5.8 \times 10^{-4}$ | 0.7 | 1.2 | 0.9 |
| 10 | 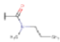 | F | $2.4 \times 10^{-8} \pm 1.0 \times 10^{-9}$ | $1.8 \times 10^{5} \pm 2.5 \times 10^{4}$ | $4.2 \times 10^{-3} \pm 5.2 \times 10^{-4}$ | $5.5 \times 10^{-8} \pm 4.4 \times 10^{-9}$ | $2.3 \times 10^{5} \pm 5.5 \times 10^{4}$ | $1.2 \times 10^{-2} \pm 2.1 \times 10^{-3}$ | 0.4 | 0.8 | 0.3 |
| 11 | 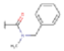 | Me | $9.8 \times 10^{-9} \pm 3.0 \times 10^{-10}$ | $1.1 \times 10^{5} \pm 1.8 \times 10^{4}$ | $1.1 \times 10^{-3} \pm 2.0 \times 10^{-4}$ | $4.0 \times 10^{-9} \pm 5.3 \times 10^{-10}$ | $8.0 \times 10^{5} \pm 7.4 \times 10^{4}$ | $3.2 \times 10^{-3} \pm 2.2 \times 10^{-4}$ | 2.4 | 0.1 | 0.4 |
| 12 | 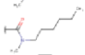 | Me | $3.8 \times 10^{-8} \pm 5.3 \times 10^{-9}$ | $2.8 \times 10^{5} \pm 7.4 \times 10^{4}$ | $1.0 \times 10^{-2} \pm 1.6 \times 10^{-3}$ | $6.7 \times 10^{-8} \pm 8.0 \times 10^{-9}$ | $5.8 \times 10^{5} \pm 6.1 \times 10^{4}$ | $3.9 \times 10^{-2} \pm 6.8 \times 10^{-3}$ | 0.6 | 0.5 | 0.3 |
| 13 | 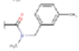 | Me | $1.6 \times 10^{-8} \pm 3.4 \times 10^{-9}$ | $7.8 \times 10^{4} \pm 1.3 \times 10^{4}$ | $1.2 \times 10^{-3} \pm 1.0 \times 10^{4}$ | $4.1 \times 10^{-9} \pm 4.2 \times 10^{-10}$ | $8.5 \times 10^{5} \pm 1.0 \times 10^{5}$ | $3.4 \times 10^{-3} \pm 1.7 \times 10^{-4}$ | 3.9 | 0.1 | 0.4 |
| 14 | 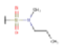 | F | $8.7 \times 10^{-8} \pm 2.1 \times 10^{-9}$ | $7.7 \times 10^{4} \pm 1.8 \times 10^{4}$ | $6.4 \times 10^{-3} \pm 4.3 \times 10^{-4}$ | $1.4 \times 10^{-8} \pm 7.5 \times 10^{-10}$ | $2.5 \times 10^{5} \pm 1.1 \times 10^{4}$ | $3.5 \times 10^{-3} \pm 1.5 \times 10^{-4}$ | 6.2 | 0.3 | 1.9 |
| 15 | 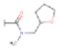 | Me | $8.9 \times 10^{-8} \pm 1.4 \times 10^{-8}$ | $5.3 \times 10^{4} \pm 1.7 \times 10^{4}$ | $4.6 \times 10^{-3} \pm 9.0 \times 10^{-4}$ | $2.1 \times 10^{-8} \pm 4.0 \times 10^{-9}$ | $1.2 \times 10^{5} \pm 2.8 \times 10^{4}$ | $2.5 \times 10^{-3} \pm 4.3 \times 10^{-4}$ | 4.2 | 0.4 | 1.8 |
| 16 | 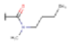 | F | $2.3 \times 10^{-8} \pm 4.4 \times 10^{-9}$ | $7.0 \times 10^{4} \pm 7.5 \times 10^{3}$ | $1.7 \times 10^{-3} \pm 4.6 \times 10^{-4}$ | $8.7 \times 10^{-9} \pm 6.2 \times 10^{-10}$ | $4.5 \times 10^{5} \pm 4.4 \times 10^{4}$ | $3.9 \times 10^{-3} \pm 3.8 \times 10^{-4}$ | 2.7 | 0.2 | 0.4 |
| 17 | 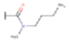 | - | $1.9 \times 10^{-8} \pm 1.4 \times 10^{-8}$ | $1.5 \times 10^{5} \pm 2.1 \times 10^{4}$ | $3.0 \times 10^{-3} \pm 2.6 \times 10^{-4}$ | $8.8 \times 10^{-9} \pm 8.4 \times 10^{-10}$ | $3.6 \times 10^{5} \pm 2.4 \times 10^{4}$ | $3.2 \times 10^{-3} \pm 1.2 \times 10^{-4}$ | 2.1 | 0.4 | 0.9 |
| 18 | 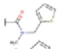 | Me | $1.1 \times 10^{-8} \pm 7.8 \times 10^{-10}$ | $1.1 \times 10^{5} \pm 1.5 \times 10^{4}$ | $1.2 \times 10^{-3} \pm 2.5 \times 10^{-4}$ | $6.2 \times 10^{-9} \pm 2.2 \times 10^{-9}$ | $2.8 \times 10^{5} \pm 3.7 \times 10^{4}$ | $1.7 \times 10^{-3} \pm 3.5 \times 10^{-4}$ | 1.8 | 0.4 | 0.7 |
| 19 | 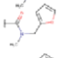 | Me | $6.1 \times 10^{-10} \pm 2.2 \times 10^{-10}$ | $6.1 \times 10^{5} \pm 1.9 \times 10^{5}$ | $3.4 \times 10^{-4} \pm 3.6 \times 10^{-5}$ | $1.7 \times 10^{-9} \pm 1.9 \times 10^{-10}$ | $1.8 \times 10^{6} \pm 1.7 \times 10^{5}$ | $3.0 \times 10^{-3} \pm 1.5 \times 10^{-4}$ | 0.4 | 0.3 | 0.1 |
| 20 | 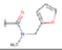 | F | $7.2 \times 10^{-10} \pm 4.0 \times 10^{-11}$ | $4.6 \times 10^{5} \pm 5.4 \times 10^{4}$ | $3.3 \times 10^{-4} \pm 2.1 \times 10^{-5}$ | $4.3 \times 10^{-9} \pm 8.5 \times 10^{-10}$ | $6.2 \times 10^{5} \pm 1.2 \times 10^{5}$ | $2.6 \times 10^{-3} \pm 1.2 \times 10^{-4}$ | 0.2 | 0.7 | 0.1 |

**Supplementary Table 4** Computed desolvation free energies of loop- and helix-binding compounds (compounds **1-6** and **7-20**, respectively), **$\Delta G_{desolv}$ (3D-RISM) -** 3D-RISM hydration free energies for the compounds computed using the MOE software [10,11] and taken with the opposite sign. The average RMS deviation of the solvation free energy computed using the 3D-RISM method from that obtained by free energy perturbation was estimated as less than about ±5.4 $kJ.mol^{-1}$ (see Ref. [12]).

| **Compounds** | **$\Delta G_{desolv}$ (3D-RISM) ($kJ.mol^{-1}$)** |
|---|---|
| 1 | 81.7 |
| 2 | 68.1 |
| 3 | 79.4 |
| 4 | 81.9 |
| 5 | 76.9 |
| 6 | 69.8 |
| 7 | 118.7 |
| 8 | 88.2 |
| 9 | 90.7 |
| 10 | 89.7 |
| 11 | 99.0 |
| 12 | 91.5 |
| 13 | 97.0 |
| 14 | 86.6 |
| 15 | 115.6 |
| 16 | 88.6 |
| 17 | 94.5 |
| 18 | 101.9 |
| 19 | 99.0 |
| 20 | 103.9 |

**Supplementary Table 5** Characteristics of crystallographic water sites in the crystal structures of NHSP90-ligand complexes. Only water sites within 0.8 nm of N106 were included in the analysis**.** $\rho^{WaTCH}$ - population of the water site in a set of crystal structures of N-HSP90-ligand complexes computed using the WaTCH program using 8 helical and 8 loop-in structures (PDB codes are given in **Supplementary Table 6**); $\triangle G^{3D\text{-}RISM}$ - calculated solvation free energy of the water site computed with the 3D-RISM approach for the complexes with compounds **1** and **16** using MOE software[10] ; only hydration sites associated with water molecules of the corresponding crystal structure (i.e. located in the vicinity) are given; $R^{3D\text{-}RISM}$ - distance to the nearest crystallographic water site which is given in the second column. The total number of stable water sites found for helix-type complexes: **8**, **16**, **14**, **18**, and **20** are 4, 6, 4, 6, and 6, respectively at the density iso-value of 1.5; 5 for both loop-type complexes with compounds **1** and **4**. $\rho^{GIST}$ - average population of the water sites with the density above water bulk density and located on the protein surface in the vicinity of α-helix3 obtained from 10 ns MD trajectories of helical and loop-in structures (see Section Methods).

The numbering of the water sites for the helical and loop-in structures corresponds to that in the PDB files of the complexes with compounds **1** and **16**, PDB IDs 5J64 and 5J9X, respectively (see also **Supplementary Figure 5**); buried sites are shown in bold. $\rho^{GIST}$ – maximum density of water molecules with respect to the bulk in the voxel computed by GIST from 10 ns MD simulations started from the crystal structures of the complexes with compounds **1, 2** and **8, 14, 16, 20**.

| **Conformation of the α-helix3 region** | **Crystallographic water site residue number in the PDB file** | $\rho_{WaTCH}$ | **$\triangle G_{3D\text{-}RISM}$ (kcal.mol$^{-1}$)** | **$R_{3D\text{-}RISM}$ (Å)** | $\rho_{GIST}$ |
|---|---|---|---|---|---|
| Loop-in | **8** | 0.87 | -9.1 | 0.5 | 6 |
| | 26 | 0.87 | -3.0 | 0.7 | 6 |
| | 59 | 0.87 | - | - | 4 |
| | **6** | 0.75 | -2.1 | 0.4 | 10 |
| | **12** | 0.75 | -2.9 | 0.6 | 4.5 |
| | 28 | 0.75 | -4.2 | 0.7 | 2.5 |
| | 48 | 0.75 | - | - | 3 |
| | 133 | 0.62 | -3.2 | 0.8 | 1.5 |
| | 122 | 0.62 | - | - | 1.5 |
| | 310 | 0.50 | - | - | - |
| Helical | **32** | 0.75 | -4.0 | 0.5 | 8 |
| | **112** | 0.50 | -2.2 | 0.6 | 12 |
| | **98** | 0.50 | -7.0 | 0.3 | 12 |
| | **102** | 0.50 | -2.7 | 0.4 | 7 |
| | **151** | 0.25 | - | - | 8 |
| | 127 | 0.25 | - | - | 3 |

**Supplementary Table 6** Crystal structures used for the analysis of water sites

| PDB code | Conformation of α-helix3 region | Resolution (Å) |
|---|---|---|
| 4CWF | helical | 2.0 |
| 4L8Z | helical | 1.7 |
| 4R3M | helical | 1.8 |
| 4LWE | helical | 1.5 |
| 4BQG | helical | 1.9 |
| 5J82 | helical | 2.2 |
| 5J9X | helical | 1.8 |
| 5J20 | helical | 1.8 |
| 4LWG | loop-in | 1.6 |
| 2BSM | loop-in | 2.05 |
| 2BT0 | loop-in | 1.9 |
| 2UWD | loop-in | 1.9 |
| 4LWH | loop-in | 1.7 |
| 4L93 | loop-in | 1.85 |
| 5J64 | loop-in | 1.4 |
| 5J2X | loop-in | 1.2 |

**Supplementary Table 7** Hsp70 up-regulation for selected compounds. For assay conditions and determination of $EC_{50}$ values, see **Methods** section.

| Compounds | $EC_{50}$ (M) |
|---|---|
| 2 | 2.9E-06 |
| 5 | 1.4E-06 |
| 9 | 1.8E-07 |
| 11 | 5.3E-08 |
| 13 | 3.2E-08 |
| 14 | 3.5E-07 |
| 15 | 1.1E-06 |
| 16 | 2.3E-07 |
| 17 | 7.7E-08 |
| 18 | 1.1E-07 |
| 19 | 6.6E-09 |
| 20 | 9.4E-09 |

**Supplementary Table 8** Sequence of primers for L107A mutant

| | Forward Primer | Reverse Primer |
|---|---|---|
| **L107A primers** | GATCTGATTAATAACGCCGGCACCATTGCAAAAAG | CTTTTTGCAATGGTGCCGGCGTTATTAATCAGATC |

## Supplementary Note 1: Supplementary Analytical Data

**1** $^{1}$H NMR (500 MHz, DMSO-d6) ppm = 11.91 (s, 1H), 9.59 (d, J=20.3, 2H), 7.43 - 7.37 (m, 1H), 7.32 - 7.24 (m, 1H), 7.23 - 7.13 (m, 2H), 7.04 (d, J=8.4, 1H), 6.22 (dd, J=8.4, 2.3, 1H), 6.14 (d, J=2.3, 1H). ESI M+H [m/z] 288.1;

**2** ESI M+H [m/z] 397.2; **3** 1H NMR (500 MHz, DMSO-d6) δ 11.86 (s, 1H), 9.62 (s, 1H), 9.60 (s, 1H), 7.29 – 7.23 (m, 2H), 7.15 – 7.11 (m, 1H), 7.02 – 6.99 (m, 1H), 6.96 – 6.93 (m, 1H), 6.17 – 6.14 (m, 2H), 2.14 (s, 3H). ESI M+H [m/z] 284.2;

**4** 1H NMR (500 MHz, DMSO-d6) δ 11.94 – 11.88 (m, 1H), 9.68 – 9.64 (m, 1H), 9.64 – 9.59 (m, 1H), 7.55 – 7.52 (m, 1H), 7.43 – 7.34 (m, 3H), 6.95 (d, J = 8.1 Hz, 1H), 6.18 – 6.14 (m, 2H). ESI M+H [m/z] 304.1;

**5** 1H NMR (500 MHz, DMSO-d6) δ 11.88 (s, 1H), 9.67 (s, 1H), 9.61 (s, 1H), 7.35 – 7.30 (m, 2H), 7.20 – 7.15 (m, 1H), 7.10 – 7.07 (m, 1H), 6.88 (d, J = 8.4 Hz, 1H), 6.17 (d, J = 2.3 Hz, 1H), 6.13 (dd, J = 8.4, 2.3 Hz, 1H), 2.49 – 2.36 (m, 2H), 1.07 (t, J = 7.6 Hz, 3H). ESI M+H [m/z] 298.1

**6** $^{1}$H NMR (400 MHz, DMSO-d6) ppm = 11.99 (s, 1H), 11.00 - 10.30 (m, 1H), 9.97 (s, 1H), 7.45 - 7.38 (m, 1H), 7.36 - 7.27 (m, 2H), 7.27 - 7.14 (m, 2H), 6.36 (s, 1H). ESI M+H [m/z] 366.0/368.0

**7** $^{1}$H NMR (500 MHz, DMSO) ppm = 12.03 (s, 1H), 10.83 (s, 1H), 10.59 (s, 1H), 7.57 - 7.51 (m, 1H), 7.50 (s, 1H), 7.47 - 7.31 (m, 3H), 6.35 (s, 1H), 3.72 (hept, J=6.7, 1H), 2.61 (s, 3H), 0.89 (d, J=6.7, 3H), 0.85 (d, J=6.6, 3H). ESI M+H [m/z] 438.9/440.9

**8** $^{1}$H NMR (500 MHz, DMSO) ppm = 12.04 (s, 1H), 10.84 (s, 1H), 10.54 (s, 1H), 7.56 (s, 1H), 7.45 - 7.39 (m, 1H), 7.32 - 7.28 (m, 1H), 7.28 - 7.23 (m, 1H), 7.19 - 7.15 (m, 1H), 6.33 (s, 1H), 3.80 (hept, J=6.7, 1H), 2.64 (s, 3H), 0.91 (d, J=6.7, 6H). ESI M+H [m/z] 423.0

**9** $^{1}$H NMR (500 MHz, DMSO) ppm = 11.91 (s, 1H), 10.04 (s, 1H), 9.92 (s, 1H), 7.30 - 7.21 (m, 2H), 7.16 - 7.09 (m, 1H), 7.02 (d, J=7.8, 1H), 6.89 (s, 1H), 6.26 (s, 1H), 3.12 (s, 2H), 2.76 (s, 3H), 2.14 (s, 3H), 1.42 (s, 2H), 0.72 (s, 3H).

**10** $^{1}$H NMR (250 MHz, DMSO) ppm = 11.95 (s, 1H), 10.05 (s, 1H), 9.88 (s, 1H), 7.47 - 7.36 (m, 1H), 7.33 - 7.13 (m, 3H), 6.95 (s, 1H), 6.25 (s, 1H), 3.25 - 3.05 (m, 2H), 2.79 (s, 3H), 1.54 - 1.36 (m, 2H), 0.82 - 0.69 (m, 3H).

**11** $^{1}$H NMR (300 MHz, DMSO) ppm = 11.89 (s, 1H), 10.15 (s, 1H), 9.94 (s, 1H), 7.39 - 7.16 (m, 7H), 7.09 - 6.92 (m, 3H), 6.28 (s, 1H), 4.60 - 4.33 (m, 2H), 2.71 - 2.62 (m, 3H), 2.13 (s, 3H). ESI M+H [m/z] 431.1

**12** $^{1}$H NMR (500 MHz, DMSO) ppm = 9.89 (s, 1H), 9.81 (s, 1H), 7.70 (d, J=1.8, 1H), 7.32 - 7.26 (m, 2H), 7.22 - 7.18 (m, 1H), 7.14 (d, J=7.7, 1H), 6.62 (s, 1H), 6.49 - 6.45 (m, 1H), 6.39 (s, 1H), 3.32 - 2.92 (m, 2H), 2.87 - 2.58 (m, 3H), 1.99 (s, 3H), 1.46 - 1.34 (m, 2H), 1.34 - 1.05 (m, 6H), 0.93 - 0.83 (m, 3H). ESI M+H [m/z] 408.3

**13** $^{1}$H NMR (400 MHz, DMSO) ppm = 11.95 - 11.83 (m, 1H), 10.22 - 10.11 (m, 1H), 10.00 - 9.91 (m, 1H), 7.27 - 7.17 (m, 3H), 7.11 - 6.94 (m, 6H), 6.27 (s, 1H), 4.57 - 4.30 (m, 2H), 2.71 - 2.63 (m, 3H), 2.30 (s, 3H), 2.12 (s, 3H). ESI M+H [m/z] 445.8

**14** $^{1}$H NMR (500 MHz, DMSO) ppm = 12.05 (s, 1H), 10.85 (s, 1H), 10.58 (s, 1H), 7.55 (s, 1H), 7.44 - 7.39

(m, 1H), 7.32 - 7.24 (m, 2H), 7.18 (td, J=7.7, 1.3, 1H), 6.34 (s, 1H), 2.94 - 2.90 (m, 2H), 2.64 (s, 3H), 1.43 (h, J=7.4, 2H), 0.80 (t, J=7.4, 3H). ESI M+H [m/z] 423.0

**15** $^{1}$H NMR (500 MHz, DMSO) ppm = 11.88 (s, 1H), 10.03 (s, 1H), 9.92 - 9.89 (m, 1H), 7.30 - 7.23 (m, 2H), 7.15 - 7.10 (m, 1H), 7.04 - 7.00 (m, 1H), 6.95 - 6.91 (m, 1H), 6.27 - 6.24 (m, 1H), 3.98 - 3.84 (m, 1H), 3.72 - 3.56 (m, 2H), 3.41 - 3.32 (m, 1H), 3.29 - 3.13 (m, 1H), 2.89 - 2.78 (m, 3H), 2.16 - 2.13 (m, 3H), 1.87 - 1.67 (m, 3H), 1.57 - 1.27 (m, 1H). ESI M+H [m/z] 425.8

**16** $^{1}$H NMR (500 MHz, DMSO) ppm = 11.95 (s, 1H), 10.05 (s, 1H), 9.88 (s, 1H), 7.46 - 7.37 (m, 1H), 7.34 - 7.13 (m, 3H), 6.96 (s, 1H), 6.25 (s, 1H), 3.20 (s, 2H), 2.79 (s, 3H), 1.43 (s, 2H), 1.20 - 1.13 (m, 2H), 0.82 (s, 3H). ESI M+H [m/z] 401.1

**17** $^{1}$H NMR (500 MHz, DMSO) ppm = 11.89 (s, 1H), 10.07 (s, 1H), 9.92 (s, 1H), 7.37 - 7.25 (m, 3H), 7.20 - 7.14 (m, 2H), 6.96 (s, 1H), 6.24 (s, 1H), 2.79 (s, 3H), 1.43 (s, 2H), 1.17 (s, 2H), 3.27 - 3.26 (m, 2H), 0.82 (s, 3H). ESI M+H [m/z] 383.1

**18** $^{1}$H NMR (500 MHz, DMSO) ppm = 11.90 (s, 1H), 10.15 (s, 1H), 9.97 (s, 1H), 7.47 - 7.45 (m, 1H), 7.27 - 7.19 (m, 2H), 7.10 - 7.04 (m, 1H), 7.03 - 6.93 (m, 4H), 6.28 (s, 1H), 4.70 - 4.52 (m, 2H), 2.77 - 2.66 (m, 3H), 2.14 (s, 3H). ESI M+H [m/z] 437.1

**19** $^{1}$H NMR (400 MHz, DMSO) ppm = 9.92 (s, 1H), 9.78 (s, 1H), 7.65 (d, J=1.8, 1H), 7.59 (dd, J=1.9, 0.9, 1H), 7.23 - 7.20 (m, 2H), 7.14 - 7.08 (m, 1H), 7.07 - 7.04 (m, 1H), 6.68 - 6.64 (m, 1H), 6.44 - 6.41 (m, 2H), 6.36 (s, 1H), 6.20 - 6.17 (m, 1H), 4.44 - 4.31 (m, 2H), 2.70 - 2.58 (m, 3H), 1.95 (s, 3H). ESI M+H [m/z] 404.1

**20** $^{1}$H NMR (500 MHz, DMSO) ppm = 11.96 (s, 1H), 10.15 (s, 1H), 9.95 (s, 1H), 7.61 - 7.60 (m, 1H), 7.42 - 7.37 (m, 1H), 7.30 - 7.25 (m, 1H), 7.22 - 7.17 (m, 1H), 7.15 - 7.11 (m, 1H), 7.02 (s, 1H), 6.42 (dd, J=3.1, 1.9, 1H), 6.29 - 6.23 (m, 2H), 4.54 - 4.38 (m, 2H), 2.77 (s, 3H). ESI M+H [m/z] 425.1

## Supplementary Methods

### Technical details of molecular dynamics simulations

The protein structure was protonated at neutral pH using Amber v15 tools[1] with all His residues treated as singly protonated on the Nδ atom, resulting in a total net protein charge of -7e (V17-G223 were included in the model). The Amber ff14SB[2] and Gaff[3] force fields for the protein and compounds, respectively, were employed in simulations. Atomic partial charges were computed for the ligands using the Restrained Electrostatic Potential (RESP) method as implemented in the R.E.D. webserver[4]. Each protein-ligand complex was solvated in a box of TIP3P water molecules extending at least 1 nm from the protein and with an additional 31 Na+ and 24 Cl- ions corresponding to a solution ionic strength of about 150mM.

Molecular dynamics (MD) simulations were performed using GROMACS 5.0.5[5] under periodic boundary conditions with a Particle Mesh Ewald treatment of long-range Coulombic interactions (Fourier spacing of 0. 12 nm and interpolation order of 4) and a non-bonded cut-off of 1 nm for Coulomb and Lennard-Jones interactions, with the latter shifted to zero beyond. Bonds to hydrogen atoms were constrained

For each protein-ligand complex, the initial system was subjected to 20000 steps of steep-descent energy minimization with all bonds constrained and then 20000 steps without constraints on nonhydrogen atoms followed by 20000 steps of conjugate gradient energy minimization. The system was heated up to 300K in steps of 10K over a total of 12ns of simulation using harmonic restraints with a force constant of 1000 kcal.mol$^{-1}$Å$^{-2}$ on all solute atoms. The system was then equilibrated for 2 ns under NVT conditions using a Berendsen thermostat (300K) with the positions of the protein and the ligand restrained using a force constant of 100 kcal.mol$^{-1}$Å$^{-2}$. After this, three MD equilibration steps of 10 ns each with restraints decreasing in each step from 100 to 50 to 10 kcal. mol$^{-1}$Å$^{-2}$ were performed at NPT conditions using a Nose-Hoover thermostat and a Parrinello-Rahman barostat keeping the temperature and pressure at 300K and 1 bar, respectively. Finally, the restraints were removed and a production trajectory was simulated with snapshots saved every 50 ps and the compressed trajectory file saved with a stride of 2 ps. A time step of 1 fs was used during the heating and 2 fs during the production simulations. Two production trajectories of about 1 µs were simulated for each complex as well as for apo-structures.

## Methods for estimation of protein conformational entropy

Various methodologies have been proposed for estimation of the conformational entropy of biomolecules from MD trajectories[6]. The first group of methods employs analysis of protein motion with a harmonic, H, or a quasi-harmonic, QH, approximation. Specifically, the harmonic approximation based on normal mode analysis, NMA, has been widely used for the estimation of the binding entropy of a ligand to a protein target, whose conformational changes upon binding are limited to side-chain rotations[7]. NMA-H computations are quite demanding and, therefore, only a small number of MD snapshots of a protein-ligand complex trajectory are usually used for these simulations, which provide the entropy arising from local fluctuations around selected conformational states represented by these snapshots. The conformational entropy of long-time scale motions can be estimated using the quasi-harmonic (QH) approximation of the protein motion. For this, principal component analysis of protein atomic (often backbone) motion is carried out. This method is very fast and relatively computationally inexpensive but, similar to the harmonic approximation, is limited to sampling of a single QH energy basin and neglects anharmonic motions and supralinear correlations between eigenmodes. As has been discussed in Ref. [8], QH method can substantially overestimates the configurational entropy of systems with multiple energy minima, especially when Cartesian coordinates are used.

The second group of methods for computation of conformational entropy directly uses configurational probability density, usually in the space of protein torsional degrees of freedom. The total conformational entropy is computed as an expansion of mutual information terms taking into account correlation effects between degrees of freedom, which generally leads to a huge configurational space to be sampled and limits application of the method to small systems. The correlation-corrected multibody local approximation, CC-MLA,[9] is one of the approximations that has been proposed to reduce the dimensionality. In this approximation, only correlation terms arising from the torsion angles of residues located within a certain distance threshold from each other are taken into account. Additionally, the computed entropy value is corrected for the negative bias of false correlation arising from incomplete sampling of degrees of freedom. Despite approximations to reduce computational costs, the CC-MLA approach still requires analysis of several hundred thousand snapshots and has been evaluated only on small molecules and peptides. It should be noted that the sampled degrees of freedom do not include global motions of the protein because only a part of the protein (α-helix1 and α-helix3) is included in simulations. Specifically, oscillations of

the entire α-helix3  and α-helix1 in the helix-type structure of WT N-HSP90 represented by the 1st mode of the PCA (see **Supplementary Fig.6 B**) and observed in the backbone RMSF (see **Supplementary Fig. 6 A**) cannot be expressed solely through the fluctuations of the torsional degrees of freedom of this protein fragment. Thus, the CCMLA approach is expected to provide a lower bound on the protein conformational entropy.

## Supplementary References


1. Case, D. A. *et al.* AMBER 2015, University of California, San Francisco.
2. Lindorff-Larsen, K. *et al.* Improved side-chain torsion potentials for the Amber ff99SB protein force field. *Proteins Struct. Funct. Bioinforma.* **78,** 1950–1958 (2010).
3. Wang, J., Wolf, R. M., Caldwell, J. W., Kollman, P. A. & Case, D. A. Development and testing of a general amber force field. *J. Comput. Chem.* **25,** 1157–74 (2004).
4. François-Yves Dupradeau, Adrien Pigache *et al.* The R.E.D. tools: advances in RESP and ESP charge derivation and force field library building. *Phys. Chem. Chem. Phys.* **12,** 7821–7839 (2010).
5. Abraham, M. J. *et al.* Gromacs: High performance molecular simulations through multi-level parallelism from laptops to supercomputers. *SoftwareX* **1–2,** 19–25 (2015).
6. Meirovitch, H., Cheluvaraja, S. & White, R. P. Methods for calculating the entropy and free energy and their application to problems involving protein flexibility and ligand binding. *Curr. Protein Pept. Sci.* **10,** 229–243 (2009).
7. Ben-Shalom, I. Y., Pfeiffer-Marek, S., Baringhaus, K.-H. & Gohlke, H. Efficient Approximation of Ligand Rotational and Translational Entropy Changes upon Binding for Use in MM-PBSA Calculations. *J. Chem. Inf. Model.* **57,** 170–189 (2017).
8. Chang, C. E., Chen, W. & Gilson, M. K. Evaluating the accuracy of the quasiharmonic approximation. *J. Chem. Theory Comput.* **1,** 1017–1028 (2005).
9. Suárez, E., Díaz, N., Méndez, J. & Suárez, D. CENCALC: A computational tool for conformational entropy calculations from molecular simulations. *J. Comput. Chem.* **34,** 2041–2054 (2013).
10. Molecular Operating Environment (MOE), 2013.08; Chemical Computing Group Inc., 1010 Sherbooke St. West, Suite #910, Montreal, QC, Canada, H3A 2R7,. (2016).
11. Application_Name, Scientific Vector Language (SVL) source code provided by Chemical Computing Group Inc., 1010 Sherbooke St. West, Suite #910, Montreal, QC, Canada, H3A 2R7,. *2016*
12. Truchon, J.-F., Pettitt, B. M. & Labute, P. A Cavity Corrected 3D-RISM Functional for Accurate Solvation Free Energies. *J. Chem. Theory Comput* **10,** 934–941 (2014).
13. Pettersen, E. F. *et al.* UCSF Chimera--a visualization system for exploratory research and analysis. *J. Comput. Chem.* **25,** 1605–12 (2004).
14. Nguyen, C. N., Kurtzman, T. & Gilson, M. K. Spatial Decomposition of Translational Water–Water Correlation Entropy in Binding Pockets. *J. Chem. Theory Comput.* acs.jctc.5b00939 (2015). doi:10.1021/acs.jctc.5b00939
15. Kleywegt, G. J. *et al.* The Uppsala Electron-Density Server. *Acta Crystallogr. Sect. D Biol. Crystallogr.* **60,** 2240–2249 (2004).
